# Risk factors for sudden cardiac death or sudden unexplained death in patients treated with clozapine: systematic review

**DOI:** 10.1192/bjo.2026.11024

**Published:** 2026-05-11

**Authors:** Tejas Easwar, Damodar Chari, Shahbaz Abdullah, Hugh Tollinton, Elizabeta B. M. Ladinska, Hari Easwar Subramaniam

**Affiliations:** School of Clinical Medicine, University of Cambridge, UK; Department of Old Age Psychiatry, Leicestershire Partnership NHS Trust, Leicester, UK; Department of Psychiatry, Leicestershire Partnership NHS Trust, Leicester, UK; School of Psychology and Vision Sciences, University of Leicester, UK

**Keywords:** Clozapine, sudden cardiac death, sudden unexplained death, risk factors

## Abstract

**Background:**

Clozapine is the gold-standard treatment for treatment-resistant schizophrenia but carries serious cardiac risks, including sudden cardiac death (SCD). Specific risk factors for SCD in clozapine-treated patients remain poorly defined.

**Aims:**

To systematically identify and synthesise evidence on risk factors for SCD and sudden unexplained death (SUD) in clozapine-treated patients, to guide clinical monitoring.

**Method:**

We conducted a systematic review following Preferred Reporting Items for Systematic reviews and Meta-Analyses guidelines (PROSPERO, no. CRD420250646384). Five databases were searched from inception to 13 October 2025. Studies reporting SCD or SUD in clozapine-treated patients were included without restrictions on study design, demographics or diagnosis. Two reviewers independently screened studies and extracted data. Quality was assessed using Joanna Briggs Institute checklists and Risk Of Bias In Non-randomised Studies of Exposures tools. Given study heterogeneity, we performed structured narrative synthesis.

**Results:**

Twenty-one studies (1989–2023) were included, comprising 498 cases of SCD/SUD in clozapine-treated patients. Risk factors were grouped into four categories: treatment intensity (high doses (≥525 mg/day), rapid titration), drug interactions (valproate, benzodiazepines, polypharmacy), lifestyle factors (smoking, obesity, diabetes, substance use) and monitoring. Two patterns emerged: early inflammatory myocarditis (weeks 2–6) and late-onset cardiomyopathy (months–years).

**Conclusions:**

Clozapine-associated SCD appears multifactorial. These findings suggest a role for gradual titration, avoidance of high-risk co-medications, baseline biomarker monitoring and ongoing management of metabolic and cardiovascular risk factors. Increased multidisciplinary surveillance may help identify patients at higher risk and inform clinical decision-making in clozapine-treated patients.

Antipsychotic medications are used widely in the treatment of psychotic disorders.^1^ Second-generation antipsychotics are generally preferred, for their efficacy and lower propensity for extrapyramidal side-effects.^2^ However, antipsychotics have been implicated in increasing the risk of sudden cardiac death (SCD).^3^ SCD is an unexpected death due to cardiac causes, occurring within a short period. SCD generally occurs within 1 h of symptom onset, primarily through mechanisms such as QT-interval prolongation leading to ventricular arrhythmias.^4^

Clozapine is widely considered a drug of choice for treatment-resistant schizophrenia (TRS).^5^ Despite its unique efficacy, clozapine’s clinical utility is constrained by several life-threatening side-effects, including agranulocytosis, seizures, myocarditis and cardiomyopathy.^6^ Although these adverse effects are well documented, the relationship between clozapine and cardiac health remains poorly understood, specifically the risk of SCD. The specific risk factors that may be associated with clozapine use remain inadequately researched. Given the importance of clozapine in TRS management and its potentially fatal cardiac complications, there is a crucial role for systematic evaluation of SCD risk in clozapine-treated patients. To address this gap, we conducted a systematic review to identify the risk factors associated with SCD in clozapine-treated patients.

## Method

### Searches

We searched five databases from inception to 13 October 2025: Medline (1946–2025), EMBASE (1947–2025), PsycINFO (1984–2025), Web of Science (1997–2025) and Scopus (1993–2025). A preliminary scoping search was conducted on 20 January 2025 to determine the feasibility of the systematic review. The protocol was then registered with PROSPERO (no. CRD420250646384) on 24 February 2025, and followed Preferred Reporting Items for Systematic reviews and Meta-Analyses (PRISMA) guidelines. Comprehensive data were extracted from the five databases on a single day (13 October 2025). The search strategy combined terms for clozapine with cardiac outcomes (QT/QTc prolongation, arrhythmias, torsades de pointes, SCD, ventricular tachyarrhythmias) and serious adverse events (paralytic ileus, agranulocytosis, sepsis, neuroleptic malignant syndrome, seizures, myocarditis, cardiomyopathy, renal failure, rhabdomyolysis) (Supplementary Information 1 available at https://doi.org/10.1192/bjo.2026.11024). Grey literature was not searched due to concerns about data quality. Reference lists of relevant papers and related PubMed articles featured were used to identify additional studies.

All searches were exported to Endnote version 21.0.1 on a macOS platform (Clarivate, London, UK; www.endnote.com),^7^ duplicates removed and the final list imported to Rayyan (Rayyan Systems Inc., Cambridge, Massachusetts, USA; www.rayyan.ai)^8^ for screening. Two reviewers (T.E., H.T.) independently screened titles and abstracts, with discrepancies resolved by a third reviewer (D.C.). All reviewers were blinded during initial screening. Full texts were independently reviewed by two reviewers (T.E., H.T.). Interrater reliability was assessed using Cohen’s kappa coefficient.

### Study selection

**Fig. 1:**
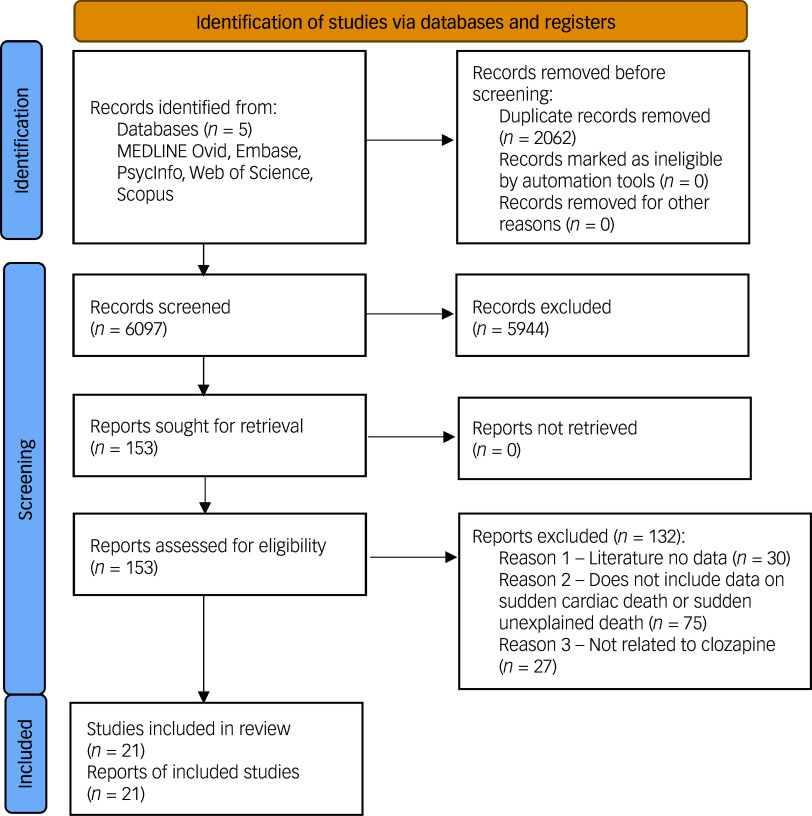
Preferred Reporting Items for Systematic reviews and Meta-Analyses flowchart.

Inclusion and exclusion criteria were agreed prior to study selection (Supplementary Information 1). We included studies where patients were treated with clozapine (either as monotherapy or in combination with other psychotropic medications) and experienced sudden unexplained death (SUD) or SCD. There were no restrictions on patients’ age, gender, diagnosis, clozapine dose, duration, formulation or route of administration. All study types were included (case series, case reports, case–control and cohort studies) except reviews and conference abstracts. Patients had to be treated with clozapine prior to the index event and could have any psychiatric condition where clozapine was prescribed. We excluded studies reporting sudden death from non-cardiac causes and overdose deaths. Reasons for exclusion are detailed in the PRISMA flowchart (Fig. 1).^9^

### Outcome definitions and ascertainment

SCD was defined as an unexpected death presumed to be cardiac in origin occurring within either 1 h of symptom onset or 24 h of last being seen well, as per standard epidemiological definitions.^10^ SUD was defined as a death with no clear cause identified, where available clinical, toxicology or post-mortem data were insufficient to attribute death to a specific pathology. Ascertainment of cause of death varied due to heterogeneity in reporting, especially in older and pharmacovigilance studies. Where autopsy findings, cardiac imaging, biomarkers or specialist cardiology assessments were available, deaths were categorised as either (a) arrhythmic SCD, (b) myocarditis-related SCD or (c) cardiomyopathy-related SCD. Deaths described using non-specific terms (such as ‘sudden death’) were conservatively classified as SUD. Studies reporting mixed or uncertain causes were included but acknowledged in the narrative synthesis, and no assumptions were made in the absence of supporting evidence.

### Quality assessment

**Fig. 2:**
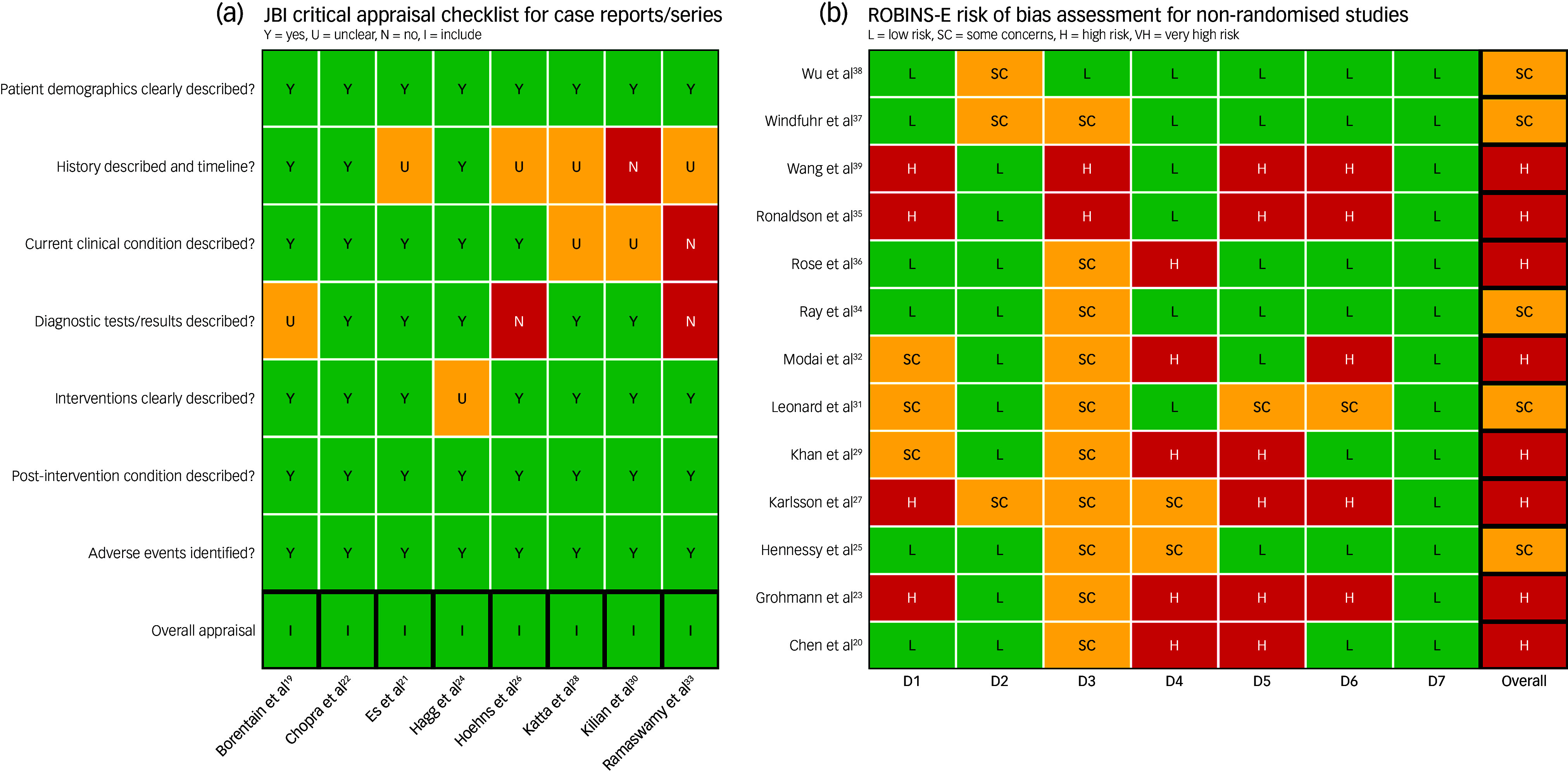
Quality assessment. JBI, Joanna Brigga Institute; ROBINS-E, Risk Of Bias In Non-randomised Studies of Exposures. ROBINS-E Quality Assessment: D1, Bias due to confounding; D2, Bias arising from measurement of the exposure; D3, Bias in selection of participants into the study; D4, Bias due to post-exposure interventions; D5, Bias due to missing data; D6, Bias arising from measurement of the outcome; D7, Bias in selection of the reported result.

We used the Joanna Briggs Institute (JBI) Critical Appraisal tools Checklist^11^ for case reports and the Risk Of Bias In Non-randomised Studies of Exposures (ROBINS-E) tool^12^ for cohort and case-control studies. ROBINS-E provides comprehensive assessment of bias risk across seven domains. Two reviewers (T.E., D.C.) independently rated each study. A traffic-light plot was created using R version 4.3.3 on a macOS platform (R Foundation for Statistical Computing, Vienna, Austria; www.r-project.org)^13^ (Fig. 2).

### Data extraction

We extracted all identifiable risk factors associated with SCD occurrence, including age, gender, psychiatric diagnosis, clozapine dose and duration, polypharmacy and physical comorbidities. Two independent reviewers (T.E., H.T.) extracted data onto Microsoft Excel sheets using a pre-designed template.^14^ Discrepancies were discussed and resolved. We extracted information on study design, sample size, demographics, psychiatric history, clozapine parameters, previous cardiac diagnoses, concomitant medications, comorbidities and statistics.

### Analysis

We initially aimed to perform a quantitative meta-analysis of risk factors; however, this was not feasible due to substantial heterogeneity in study designs, statistical reporting and outcome definitions. Consequently we undertook structured narrative synthesis adapted from the Navigation Guide systematic review framework.^15,16^ Two reviewers (T.E, D.C.) independently extracted potential risk factors and clustered them into thematic domains through discussion. Any discrepancies were resolved by consensus. Strength of evidence for each risk factor was assessed qualitatively using a modified GRADE-informed framework, considering study design, consistency, magnitude and precision of effect estimates, biological plausibility and risk of bias, consistent with established guidance for observational evidence.^17^ GRADE categories (high, moderate, low and very low) were applied to a body of evidence rather than to individual studies. Two reviewers (T.E, D.C.) independently assessed the strength of evidence for each risk factor.

## Results

### Search results

Following removal of duplicates, our search identified 6097 potentially relevant articles from five databases. Having screened titles and abstracts, 5944 articles were excluded: 333 were review articles, 14 were letters of notes and 5597 failed to meet inclusion criteria or lacked relevance to the research question. Common exclusion reasons included the following: no primary data reported; irrelevant study population; clozapine not mentioned; outcome did not include SCD/SUD; and non-English publications.

Following abstract screening, 17 conflicts were resolved through discussion. Subsequently, 153 articles underwent full-text assessment. One paper had a discrepancy on full-text review^18^ due to not fitting SCD criteria, and was excluded. We finally included 21 studies in this review.^19–39^ The PRISMA flowchart (Fig. 1) elucidates the reason for non-exclusion of studies. Interrater reliability was Cohen’s *κ* = 0.92 for full-text and abstract screening.

### Study characteristics

The final cohort comprised 21 studies spanning the period 1989–2023, representing diverse regions including the UK, Europe, North America, Australia and Asia (Table 1). Study designs included case reports (*n* = 6),^19,21,22,26,28,33^ case series (*n* = 3),^24,30,35^ retrospective cohort studies (*n* = 7),^25,27,31,32,34,36,39^ case-crossover (*n* = 2),^20,38^ case–control,^37^ prospective cohort studies (*n* = 1)^29^ and a pharmacovigilance study.^23^ Sample sizes ranged from single-patient case reports to large national database analyses involving over 200 000 patients exposed to clozapine (Table 1).

**Table 1:** Included studies

Study	Design and setting	*N*	Patient profile (age, gender)	Clozapine (dose, duration)	Likely cause of death	Notes
Borentain et al^19^	Case report (France)	1	37% male (Caucasian)	150 mg/day, 9 days	SCD	Case 2: cardiac arrest during sleep. Concomitant fluphenazine, zuclopentixol, valproate, ECT, oxazepam (300 mg/day)
Chopra and de Leon^21^	Autopsy case report (USA)	1	50% male (African American)	350 mg/day (titrated over 14 days, total 1625 mg over 14 days)	SCD-related myocarditis	Lymphocytic myocarditis (occasional eosinophils) on autopsy; concomitant valproate, quetiapine, rapid clozapine titration
Es et al^22^	Autopsy case report (Turkey)	1	48% female (Turkish)	350 mg/day (25–350 mg over 30 days; died on day 35)	SCD-related myocarditis	Eosinophilic myocarditis on autopsy; on haloperidol, biperiden, high BMI, 735 ng/mL clozapine in blood
Hoehns et al^26^	Case report (USA)	1	26% male (Caucasian)	200 mg/day (100 mg twice daily; 4 years)	SCD-related cardiomyopathy and arrhythmia	Idiopathic cardiomyopathy and underlying CAD (50–60% LAD) noted; also on risperidone, sertraline
Katta et al^28^	Autopsy case report (USA)	1	54% male (Caucasian)	500 mg/day (titrated to 500 mg; 1 year)	SCD-related myocarditis	Hypersensitivity myocarditis; on lorazepam
Ramaswamy et al^33^	Case report (USA)	1	31% male (Caucasian)	500 mg/day (titrated to 500 mg; 1 year)	SCD-related arrhythmia	Background of hypertensive heart disease with cardiac hypertrophy and chamber dilatation; on lorazepam
Hagg et al^24^	Case series (Sweden)	3	24, 26, 33% male	400–600 mg/day (8 days–20 months)	SCD-related myocarditis	All had fatal myocarditis; one patient had additional cyproterone acetate
Kilian et al^30^	Case series (Australia)	3	27–46% male	200–300 mg/day (titrated, 11–18 days)	SCD-related myocarditis	Three; all eosinophilic myocarditis on autopsy
Ronaldson et al^35^	Case series (Australia, TGA)	8	Mean 40, 60% male	Not recorded	SCD-related myocarditis	Most had minimal coronary disease on autopsy
Chen et al^20^	Case-crossover (Taiwan)	16	Not recorded	Not recorded	SCD	Clozapine use not significantly associated (aRR 0.78, 0.40–1.52)
Grohmann et al^23^	Drug surveillance (Germany)	1	31% female	200 mg/day (730 days)	SUD	No autopsy, cause unclear; clozapine + haloperidol combination suspected, previous inter-atrial surgery 13 years previously
Hennessy et al^25^	Cohort (US Medicaid)	19	Mean 40%, 40% female	Various (not recorded)	SCD- and SCD-related arrhythmia	Overall clozapine rate lower than haloperidol/risperidone (non-significant)
Karlsson et al^27^	Cohort (USA, Vigibase)	235	Median 42%, 32% female	Not recorded	SCD	235 of 462 SCDs on atypicals occurred in clozapine users (significant over-representation)
Khan et al^29^	Prospective cohort (Australia)	10	Mean 47%, 90% male	Average level 439 ng/mL; 5-year follow-up	SCD- and SCD-related arrhythmia	29 deaths: 10 sudden (6%), 14 myocarditis (3%); half (5) of sudden deaths had ventricular arrhythmia
Modai et al^32^	Cohort (Israel)	6	Not recorded (all had schizophrenia)	Not recorded	SUD	CTPs had 3.8× higher rate of sudden death versus non-CTPs (*z* = 2.48, *P* < 0.05)
Ray et al^34^	Cohort (USA, Medicaid)	4	Mean 45.7%, 35% male	Not recorded	SCD	IRR 3.67 (1.94–6.94, *P* < 0.001) CTPs versus non-CTPs
Rose et al^36^	Case–control (UK trust)	17	Mean 51.9%, 76.5% male	Mean 460 mg/day	SUD	40% were on >525 mg; many had additional psychotropics (antipsychotics, antidepressants, mood stabilisers)
Wang et al^39^	Autopsy series (China)	13	Not recorded	Not recorded	SUD and SCD	Six SUD cases, seven SCD cases; limited case details available
Windfuhr et al^37^	Case–control (UK)	16	Not recorded	Not recorded	SUD	Clozapine over-represented
Wu et al^38^	Case–crossover (Taiwan)	141	Not recorded	Not recorded	SCD- and SCD-related arrhythmia	Clozapine associated with SCD risk; aOR 2.03 (95% CI 0.83–4.93); limited details about clozapine demographics
Leonard et al^31^	Cohort (USA, Medicaid)	Not recorded	Not recorded	Not recorded	SCD- and SCD-related arrhythmia	SCD/VA; clozapine had lower adjusted hazard versus olanzapine (hazard ratio 0.58, 0.39–0.89)

SCD, sudden cardiac death; ECT, electroconvulsive therapy; BMI, body mass index; CAD, coronary artery disease; LAD, left anterior descending artery; TGA, therapeutic goods administration; aRR, adjusted relative risk; SUD, sudden unexpected death; CTP, clozapine-treated patients; IRR, incidence rate ratio; aOR, adjusted odds ratio; VA, ventricular arrhythmia.

### Quality assessment

Overall quality of the included case reports was high, but risk of bias among larger observational studies was heterogenous (Fig. 2). Evaluation with the JBI checklist showed that most case reports adequately described patient demographics, interventions and outcomes, although a minority omitted details on timelines,^26,28,30,33^ current clinical description^28,30,33^ and diagnostic testing.^19,23,26,33^ Using ROBINS-E to assess 13 non-randomised studies, 8 were judged to be at high risk of bias^20,23,27,29,32,35,36,39^ and 5 raised some concerns.^25,31,34,37,38^ The limitations for each risk factor domain are summarised in Supplementary Table 1. Overall, these limitations highlight the importance of cautious interpretation of causal inferences from these studies.

### Patient characteristics

Across all studies, 498 patients treated with clozapine experienced SCD or SUD, with baseline characteristics identified (Table 2). Most studies involved adult patients with schizophrenia or schizoaffective disorder, although some included mixed psychiatric diagnoses. Most cohorts were predominantly male: the mean proportion of males was 68% (95% CI 62.8–73.3%). Participant ages ranged from 24 to 54 years, with a mean of 36.94 years (s.d. 9.86). Myocarditis, cardiomyopathy and ventricular arrhythmia were reported as direct causes of death in a subset of cases. Several studies included comparator groups of patients receiving other second-generation antipsychotics, typical antipsychotics or no antipsychotic exposure (Table 3 **)**.

**Table 2:** Characteristics of the included cohort

Characteristics	Statistics
Mean age, years (s.d.)	36.94 (9.86), range 24–54
Proportion male (95% CI)	68.0 (62.8–73.3%), range 58.4–90.0%
Mean clozapine dose (s.d.)	320.82 (157.33) mg/day, (range 100–600 mg/day)
Mean duration of clozapine treatment, days (s.d.)	549.2 (867.26), (range 8–2920)
Sudden cardiac death (SCD)	90.76% (452/498)
Sudden unexpected death	9.23% (46/498)
Myocarditis-related SCD	3.41% (17/498)
Cardiomyopathy-related SCD	0.2% (1/498)
Arrhythmia-related SCD	1.41% (7/498)

**Table 3:** Risk of sudden cardiac death for clozapine compared with other antipsychotics

Study	Comparator	Clozapine	Notes
Wu et al^38^	All antipsychotics, odds ratio 1.53 (1.38–1.70)	Odds ratio 2.03 (0.83–4.94)	Not the highest-risk drug (clothiapine and clopenthixol had a higher odds ratio)
Hennessy et al^25^	Haloperidol	Sudden cardiac death (SCD) rate, 2.2/1000 person-years (1.3–3.4)	Lower SCD risk than haloperidol, but not statistically significant; adjusted risk ratio 0.7 (0.4–1.2)
Windfuhr et al^37^	Atypical antipsychotic, odds ratio 1.28 (0.92–1.80)	Odds ratio 2.16 (1.03–4.55)	Polypharmacy (odds ratio 2.35), promazine higher odds (odds ratio 4.02, 1.33–12.14)
Modai et al^32^	Non-clozapine-treated patients	Risk ratio 3.8	CTPs who had sudden death were younger and healthier than non-CTPs
Ray et al^34^	Any atypical antipsychotic, incidence rate ratio 2.26 (1.88–2.72)	Incidence rate ratio 3.67 (1.94–6.94, *P* < 0.001)	Clozapine had the highest incidence rate ratio among all typical and atypical antipsychotics

CTP, clozapine-treated patients.

### Risk factors

**Fig. 3:**
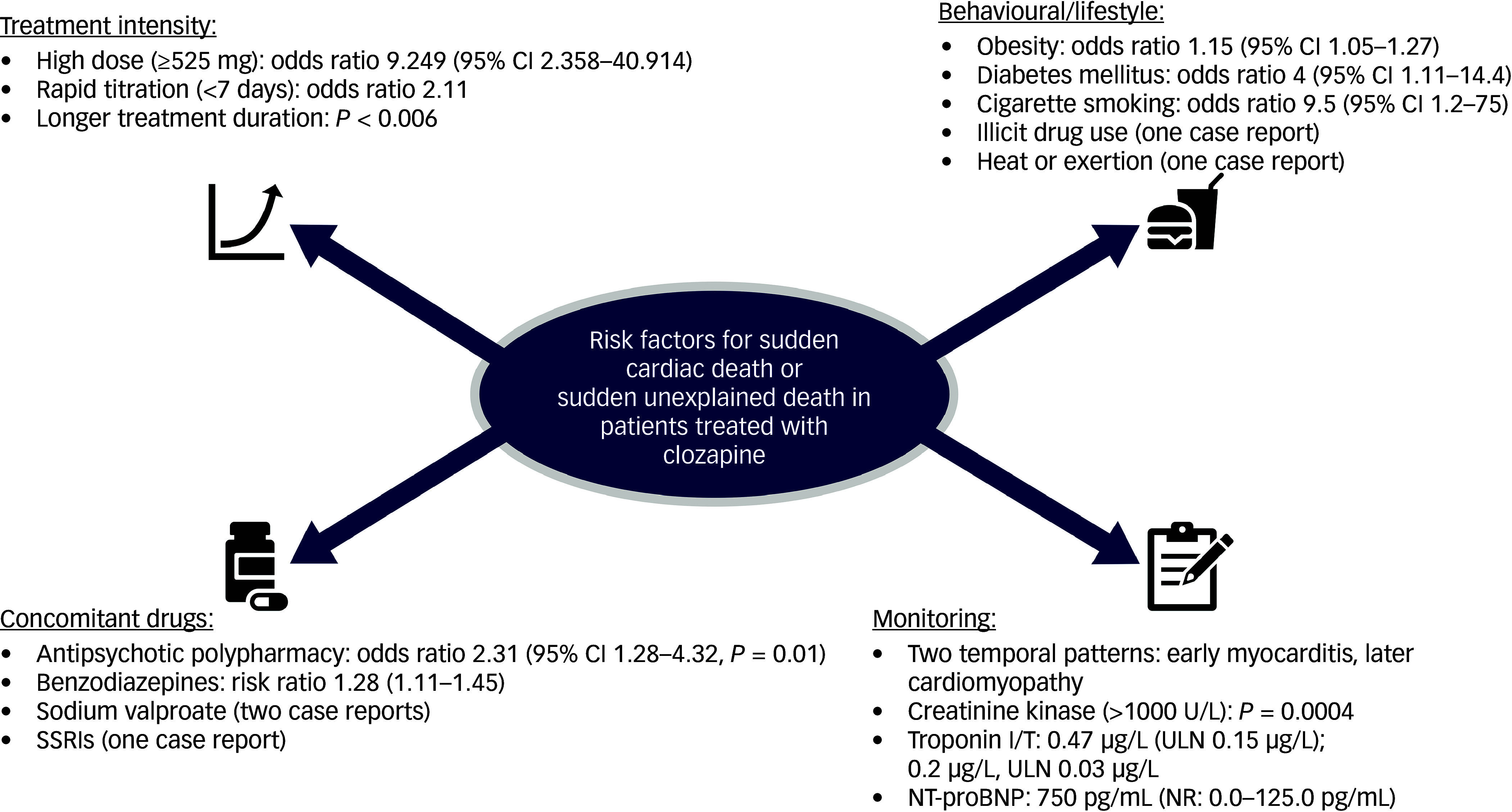
Summary of risk factors. SSRIs, selective serotonin reuptake inhibitors; ULN, upper limit of normal; NT-proBNP, N-terminal pro-B-type natriuretic peptide.

Structured narrative synthesis identified four main domains of risk factors for SCD and SUD in clozapine-treated patients: treatment intensity, concomitant drug interactions, monitoring and behavioural/lifestyle factors (Table 4 and Fig. 3). Treatment intensity refers to clozapine administration and dosing. We identified high daily dose, rapid titration and cumulative exposure as risk factors. Concomitant drug interactions refer to polypharmacy and specific drug combinations. We identified polypharmacy, sodium valproate and benzodiazepines as key risk factors. Behavioural/lifestyle factors (daily habits, actions and ways of living) were also found to modulate risk of SCD in clozapine-treated patients. We identified metabolic risk factors, including obesity, diabetes mellitus and cigarette smoking. Monitoring refers to the use of clinical, laboratory or imaging tests for the early detection or tracking of myocarditis or other cardiac damage. We observed troponin I/T and creatinine kinase to be raised in a clozapine fatal myocarditis case in a series by Ronaldson et al,^35^ and N-terminal pro-B-type natriuretic peptide (NT-proBNP) in a case by Katta et al.^28^

**Table 4:** Major risk factor categories identified among clozapine-treated patients with sudden cardiac death (SCD) or sudden unexplained death (SUD), and supporting evidence

Category	Risk factors	Supporting evidence – modified GRADE framework	Notes
Treatment intensity	High clozapine dose (≥525 mg/day)	Medium – one retrospective cohort study (SUD): Rose et al^36^	Odds ratio 9.249 (95 CI 2.358–40.914) for ≥525 mg
	Rapid dose titration	Medium – one case-crossover study (SCD) and one case report (SCD-related myocarditis): Wu et al^38^, Chopra et al^21^	Odds ratio 2.11 (patients treated with antipsychotics <7 days)
	Long treatment duration	Low – one case series (SCD-related myocarditis), but statistical test: Ronaldson et al^35^	*P* < 0.006 (mean 20.8 (14–33) days *v*. 17 (10–26) days for fatal versus non-fatal)
Concomitant medications	Sodium valproate	Low – two case reports (SCD-, SCD-related myocarditis): Borentain et al^19^, Chopra et al^21^	–
	Benzodiazepines (e.g. diazepam)	Medium – one case-crossover study (SCD) and two case reports (SCD-, SCD-related arrhythmia): Chen et al^20^, Borentain et al^19^, Ramaswamy et al^33^	Crude risk ratio 1.28 (1.11–1.45, *P* < 0.001)
	Multiple antipsychotics (polypharmacy)	Medium – one case–control study (SUD): Windfuhr et al^37^	Odds ratio 2.31 (95% CI 1.28–4.32, *P* = 0.01)
	Selective serotonin reuptake inhibitors (e.g. sertraline)	Very low – one case report (SCD): Hoehns et al^26^	Suggested that selective serotonin reuptake inhibitors may elevate clozapine levels via CYP interactions
Behavioural/ lifestyle	Obesity (high body mass index)	Medium – one prospective cohort study (SCD), one retrospective cohort study (SUD), one case series and one case report (SCD-related myocarditis): Khan et al^29^, Modai et al^32^, Ronaldson et al^35^, Es et al^22^	Odds ratio 1.15 (95% CI 1.05–1.27, *P* = 0.003) *P* < 0.03 (60 *v*. 26% for fatal versus non-fatal) Body mass index 42.73 (Es et al^22^)
	Diabetes mellitus	Medium – one prospective cohort study (SCD): Khan et al^29^	Odds ratio 4 (95% CI 1.11–14.4, *P* = 0.04)
	Cigarette smoking	Medium – one prospective cohort study (SCD): Khan et al^29^	Odds ratio 9.5 (95% CI 1.2–75, *P* = 0.03)
	Illicit drugs	Very low – one prospective cohort study but no statistical test (SCD): Khan et al^29^	There were 7 out of 10 cases with sudden death: cannabis (*n* = 4), amphetamines (*n* = 2), heroin (*n* = 1)
	Heat or exertion	Very low – one retrospective cohort study but no statistical test (SUD): Rose et al^36^	High proportion on hot days (heatwave)
Monitoring	↑Troponin I/T	Very low – one case series, two cases (SCD-related myocarditis): Ronaldson et al^35^	Troponin I 0.47 µg/L (upper limit of normal (ULN) 0.15 µg/L); Ttoponin T 0.2 µg/L (ULN 0.03 µg/L)
	↑ Creatine kinase	Low – one case report but statistical test (SCD-related myocarditis): Ronaldson et al^35^	Creatinine kinase >1000 U/L (*P* = 0.0004) in fatal cases
	↑ N-terminal pro-B-type natriuretic peptide (NT-proBNP)	Very low – one case report (SCD-related myocarditis): Katta et al^28^	NT-proBNP 750 pg/mL (0–125.0 pg/mL)

## Discussion

This systematic review synthesises current evidence on risk factors for SCD or SUD in clozapine-treated patients from 21 studies spanning the period 1989–2023. Our findings build on prior meta-analysis, reporting a threefold increase in SCD risk with clozapine (odds ratio 3.67, 95% CI 1.94–6.94), the highest risk among antipsychotics except thioridazine.^3^ Global pharmacovigilance data from the World Health Organization corroborate these concerns, identifying SCD and SUD as the second leading cause of death in clozapine-treated patients behind pneumonia, with 1449 fatal cases recorded from inception to July 2019.^40^ However, a recent VigiBase analysis highlights important limitations of pharmacovigilance data, noting that the most frequently reported fatal outcome was the non-specific category ‘death’, which may misattribute causative adverse drug reactions.^41^ We identified four main risk factor domains: treatment intensity (high doses, rapid titration), drug interactions (valproate, benzodiazepines, polypharmacy), lifestyle/behavioural factors (smoking, obesity, diabetes, substance use) and monitoring considerations. This multifactorial risk profile highlights the importance of vigilant short- and long-term monitoring in patients treated with clozapine.

### Treatment intensity

Studies reported higher doses and rapid titration among SUD and SCD cases, consistent with a prior systematic review linking higher doses with myocarditis and cardiomyopathy.^42^ Whereas British National Formulary (BNF) guidelines advise slow titration in steps of 50–100 mg once or twice weekly to a maximum daily dose of 200–450 mg over 14–21 days,^43^ contemporary evidence supports a more individualised approach. The Maudsley Prescribing Guidelines recommend titration tailored to gender and smoking status; slower titration in female non-smokers compared with male smokers, reflecting metabolic differences; and suggest CYP1A2 pharmacogenetic testing to identify patients requiring lower doses.^44^ Observational data further link aggressive early dose escalation with myocarditis: an Australian case–control study reported a 26% increase in myocarditis risk for each additional 250 mg added in the first 9 days of treatment.^45^ Multiple case reports in this review described accelerated titration rates preceding SCD.^19,21,24^ Collectively, these findings suggest the need for personalised, biomarker-informed, cautious clozapine initiation. What needs to be further established is whether enhanced monitoring is required for those on higher doses.

### Concomitant drug interactions

Our finding of valproate co-prescription in two case reports of SCD and myocarditis-related SCD^19,21^ aligns with meta-analyses identifying increased risk of clozapine-related myocarditis and cardiomyopathy (pooled odds ratio 3.58, 95% CI 1.81–7.06).^42^ One pharmacokinetic study suggested that valproate inhibits clozapine metabolism, with a mean plasma clozapine increase of 22.5%.^46^ A cohort study found that concomitant valproate during clozapine initiation increased inflammatory adverse events, including myocarditis (odds ratio 3.25, 95% CI 1.03–9.74).^47^ It is perceived that clozapine-related myocarditis occurs at least partly as a result of an inflammatory reaction,^48^ and co-factors such as valproate that raise clozapine exposure or provoke inflammation may increase this risk.

By contrast, our finding of benzodiazepine co-prescription in two case reports of SCD^19,33^ has sparse direct evidence in clozapine cohorts. A retrospective New York cohort of 152 patients co-prescribed clozapine and benzodiazepine reported no cardiac arrests or sudden deaths.^49^ However, broader cardiology literature describes increased SCD incidence following benzodiazepine initiation (adjusted hazard ratio 2.01, 95% CI 1.42, 2.83),^50^ suggested as reflecting respiratory depression and secondary arrhythmias.^51,52^

Sertraline, a selective serotonin reuptake inhibitor (SSRI), was identified in a single case report of SCD,^26^ although pooled meta-analysis has not shown an increased SCD risk with SSRIs overall (odds ratio 0.36, 95% CI 0.038–1.600).^53^ One large pharmacokinetic study reported a negative correlation between sertraline dose and clozapine concentration (*r*s = −0.535, *P* = 0.048).^54^ However, some studies have shown that SSRIs can have cardiovascular side-effects including fatal arrhythmias, often through QT-interval prolongation.^55–57^

### Lifestyle and behavioural factors

We identified metabolic comorbidities, including obesity and diabetes mellitus, as potential contributors to SCD and SUD.^22,29,32,35^ Pharmacokinetic studies show that body mass index (BMI) and glycaemic abnormalities affect clozapine metabolism through greater deposition in adipose tissue and influence on hepatic enzyme activity, resulting in higher exposure in some patients.^58–60^ Meta-analyses show that diabetes and obesity independently increased SCD risk (risk ratio 2.02, 95% CI 1.81–2.25 and risk ratio 1.16, 95% CI 1.05–1.28, respectively),^61,62^ whereas clozapine itself causes rapid weight gain and dysglycaemia, further exacerbating cardiac risk,^63^ with obesity being associated with increased blood levels of clozapine.^64^ Accordingly, international guidance advises personalised slower titration for patients with high BMI or other clinical vulnerabilities.^65^

We identified cigarette smoking as a contributor to SCD in one cohort,^29^ appearing to amplify cardiovascular risk. One meta-analysis found that current smokers have a threefold increased risk of SCD than never-smokers (risk ratio 3.06, 95% CI 2.46–3.82).^66^ Mechanistically, tobacco smoke induces the liver enzyme CYP1A2,^67^ producing significantly lower clozapine plasma concentration levels (standard mean difference −0.39, 95% CI −0.55 to −0.22, *P* < 0.001).^68^ Similarly, a retrospective cohort study reported clinically important rises in clozapine levels following abrupt smoking cessation.^69^ This could be explained by CYP1A2 activity declining following smoking cessation.^70^ However, this review could not find any association of smoking cessation with SCD or SUD. Closer therapeutic drug monitoring and dose adjustments following smoking cessation warrant further investigation.

We also identified an increased prevalence of illicit drug use (including cannabis, opioids and stimulants) with SCD in one cohort,^29^ although the study had insufficient power for statistical analysis. A recent meta-analysis showed that cannabis was associated with increased cardiovascular (risk ratio 2.10, 95% CI 1.29–3.42),^71^ chronic opioid exposure with increased CVD risk (pooled odds ratio 1.74, 95% CI 1.12–2.70)^72^ and amphetamines with higher cardiovascular mortality (odds ratio 5.12, 95% CI 3.74–7.00).^73^ Stimulants produce catecholamine surges, coronary vasospasms and direct myocardial toxicity that may precipitate ventricular arrhythmia and promote chronic dilated cardiomyopathy.^74,75^

### Monitoring

Two temporal patterns of clozapine-associated SCD were identified: an early-onset inflammatory myocarditis and later-onset cardiomyopathy, consistent with a previous review.^76^ In a fatal myocarditis-related SCD case series, Ronaldson et al^35^ reported elevated troponin I (TnI) and T (TnT) in two affected patients. Cardiac troponins are cardiomyocyte-specific regulatory proteins released following myocardial injury, extensively validated as biomarkers for myocardial infarction.^77,78^ An Australian case control study reported that 90% of patients with clozapine myocarditis had TnI or TnT concentrations that exceeded twice the upper limit of normal (ULN).^79^ ULN represents the assay-specific maximum threshold, typically defined as the 99th percentile of a healthy reference population. Reported ULNs for TnI and TnT in this study ranged from 0.03 to 0.60 μg/L.^79^ Similarly, data from a London (UK) electronic health registry showed strong predictive performance of troponin-based models in the detection of clozapine-associated myocarditis, with an area under the receiver operating characteristic curve (AUC) of 0.975 in logistic regression analysis.^80^ Given that AUC values range from 0.5 (no discriminative ability) to 1.0 (perfect discrimination), an AUC of 0.975 reflects excellent accuracy in distinguishing patients with clozapine-induced myocarditis. In broader cardiology cohorts, a large Scottish study found that TnI was more strongly associated with cardiovascular disease (CVD) and CVD mortality, whereas TnT was associated more with non-CVD-related deaths.^81^ These findings suggest that TnI may offer better specificity for clozapine-related myocarditis and SCD; however, this requires further validation in dedicated psychiatric cohorts.

Creatinine kinase elevation was also seen with myocarditis-related SCD in the study of Ronaldson et al.^35^ Creatinine kinase is involved in cellular energy metabolism, with its myocardial isoenzyme CK-MB historically used in the diagnosis of myocardial infarction before being replaced by troponins.^82^ A post-mortem meta-analysis showed significantly higher CK-MB in SCD patients (standard mean difference 0.63, 95% CI 0.09–1.17, *P* = 0.02).^83^ A prospective study in Georgia (USA) involving 100 patients (4 developed myocarditis) reported creatinine kinase abnormalities in 30% of first-exposure and 46% of re-initiated patients – considerably higher than troponin I (3.5 and 0%, respectively) or C-reactive protein (CRP) (23 and 39%, respectively),^84^ suggesting that creatinine kinase elevation is common but poorly specific for myocarditis. Rises can occur secondary to non-cardiac factors such as rhabdomyolysis, strenuous exercise or trauma.^85^

CRP, also identified with myocarditis-related SCD,^35^ is an acute-phase inflammatory marker.^86^ One meta-analysis showed that higher CRP concentrations carried a greater risk of sudden death (hazard ratio 1.19, 95% CI 1.09–1.29).^87^ Although we did not observe a direct association between CRP and clozapine SCD, a retrospective cohort reported excellent discrimination (AUC 0.876) for clozapine-related myocarditis.^80^ However, CRP elevation lacks specificity for clozapine-related myocarditis and may be raised in concurrent infection, inflammation, comorbidities and other factors.^76^ Despite these limitations, in a proposed monitoring protocol, baseline measurements of troponin, CRP and echocardiography are recommended, with clozapine cessation if troponin exceeds twofold ULN or CRP >100 mg/L.^79^ Similarly, the Maudsley Prescribing Guidelines recommend weekly monitoring of CRP, creatinine kinase and troponin during the first 4 weeks of treatment, or if body temperature exceeds 38°C, with elevated CRP and troponin prompting further assessment using creatinine kinase, B-type natriuretic peptide and echocardiography to confirm or exclude myocarditis.^44^

NT-proBNP was raised in one case report of myocarditis-related SCD.^28^ NT-proBNP is released from cardiomyocytes in response to myocardial wall stress, and is validated for heart failure diagnosis.^88^ A cross-sectional study of 38 patients receiving chronic clozapine treatment identified subclinical left ventricular dysfunction in a minority, with NT-proBNP showing strong discriminatory performance (AUC = 0.87).^89^ Collectively, these data support troponin, CRP and NT-proBNP as useful adjunctive tests, but large prospective validating specific thresholds and efficacy are lacking for clozapine SCD and other cardiovascular side-effects.

### Distinct mechanistic pathways of SCD

Evidence from the included studies suggests at least two partially distinct pathways of SCD in clozapine-treated patients, with implications for monitoring and prevention. An inflammatory-structural pathway involves early-onset myocarditis, sometimes progressing to dilated cardiomyopathy, typically within weeks to months of treatment, with highest risk during early exposure, rapid titration and concomitant sodium valproate use.^21,24,28,30,35,42^ By contrast, an electrophysiological pathway may involve QT prolongation, autonomic instability or ventricular arrhythmias leading to SCD without preceding structural disease.^3,25,38^ These differing mechanisms may suggest the need for different preventive strategies.

### Clinical implications

Currently in the UK, the National Institute for Clinical Excellence and BNF advise baseline cardiac assessment before starting clozapine; specialist review if abnormalities are present; discontinuation if myocarditis or cardiomyopathy is suspected; and ongoing cardiovascular risk monitoring, including electrocardiogram review, in patients with cardiovascular risk factors.^43,90^ However, a previous systemic review highlighted the limited high-quality evidence for intensive routine cardiac monitoring, alongside concerns regarding cost-effectiveness, service capacity and barriers to clozapine access.^91^ In this context, our findings highlight the rationale for considering refinement rather than escalation of monitoring, favouring a risk-stratified and mechanism-informed approach. Specifically, our findings support consideration of more cautious clozapine titration, avoiding rapid dose titration in early treatment and particular care in patients receiving polypharmacy. Monitoring of clozapine plasma levels in individuals at elevated risk may provide contextual pharmacokinetic information; however, supporting evidence is largely limited to clozapine-associated myocarditis,^92^ but evidence linking plasma levels with SCD or SUD remains limited. Early measurement of inflammatory and cardiac biomarkers (CRP and high-sensitivity troponin) in the first few weeks of initiation has been proposed for clozapine-associated myocarditis; however, evidence that such strategies reduce SCD or SUD is lacking, and larger-scale trials are required. Routine cardiovascular risk management, including smoking cessation support, substance misuse management, careful dose adjustment and regular review of high-risk co-medications (particularly valproate and multiple antipsychotics), is important for safe clozapine prescribing but has limited direct evidence for SCD or SUD reduction. Emerging digital and wearable technologies may have a future role in longer-term risk monitoring, although evidence remains preliminary.^93^ Importantly, these risks must be balanced against clozapine’s substantial survival benefits: an important meta-analysis showed a significant reduction in all-cause mortality despite cardiometabolic concerns (risk ratio 0.43, 95% CI 0.34–0.55), and clozapine was the largest protective factor for suicide-related mortality in schizophrenia (risk ratio 0.21, 95% CI 0.15–0.29).^94^ As the only antipsychotic with proven efficacy in treatment-resistant schizophrenia, clozapine is often under-utilised due to safety concerns. Recognition of cardiac risk factors should inform proportionate cardiovascular assessment and tailored monitoring strategies, thereby supporting safe prescribing and improved access, rather than discouraging its use.

Clozapine-associated SCD and SUD appear multifactorial, involving treatment factors, patient comorbidities and pharmacological and lifestyle factors. Future studies should clarify the longer-term effectiveness and cost-effectiveness of these added monitoring strategies needed to address sudden deaths in this vulnerable population.

### Strengths and limitations

Strengths include comprehensive database searches, inclusion of broad study designs – from case report to large cohort analyses – and structured synthesis organising risk factors into clinically actionable themes. However, the evidence base is limited by the predominance of case reports and small observational studies, inconsistent definitions of SCD, SUD and myocarditis with variable autopsy rates, heterogenous reporting and an overall high risk of bias, which collectively prevent meta-analysis for most risk factors. Ethnicity was reported in some case reports but was absent from all included cohort studies, limiting assessment of ancestry-related differences in clozapine pharmacokinetics. Evidence suggests that individuals of Asian or Indigenous American ancestry have slower metabolism and increased risk of toxicity,^65^ although this review was unable to evaluate these factors. In addition, the exclusion of grey literature and non-English studies may have led to an under-ascertainment of rare but clinically important adverse effects. Consequently, our findings emphasise pattern recognition and hypothesis generation rather than definitive causal inferences. These limitations highlight the need for well-designed prospective studies and improved pharmacovigilance systems to better characterise clozapine-associated SCD.

## Supporting information

10.1192/bjo.2026.11024.sm001 Easwar et al. supplementary material 1 Easwar et al. supplementary material

10.1192/bjo.2026.11024.sm002 Easwar et al. supplementary material 2 Easwar et al. supplementary material

## Data Availability

Data availability is not applicable to this article because no new data were created or analysed in this study. The search strategy is available in Supplementary Information 1.

## Supplementary material

The supplementary material is available online at https://doi.org/10.1192/bjo.2026.11024
