# Supplementary material for: Risk factors for sudden cardiac death or sudden unexplained death in patients treated with clozapine: systematic review
Source: BJPsych Open. 2026 May 11;12(3):e130. doi: 10.1192/bjo.2026.11024 (PMC13169053; doi:10.1192/bjo.2026.11024)
Supplement: Easwar et al. supplementary material 1 — Easwar et al. supplementary material [file S2056472426110242sup001.docx]

# **Clozapine and Sudden Cardiac Death – Supplementary Information**

Table of Contents

[Clozapine and Sudden Cardiac Death – Supplemental Information 1](#_Toc208344526)

[Search Strategy 2](#_Toc208344527)

[**Medline and EMBASE Search Strategy via Ovid using MeSH Terms** 2](#_Toc208344528)

[**Psycinfo via EBSCOhost** 9](#_Toc208344529)

[**Web of Science** 11](#_Toc208344530)

[**Scopus** 12](#_Toc208344531)

[Search Criteria 12](#_Toc208344532)

[Inclusion Criteria 12](#_Toc208344533)

[Exclusion Criteria 13](#_Toc208344534)

## **Search Strategy**

Overall Strategy:

#1 Clozapine

#2 (QT OR QTc OR arrhythmia* OR torsad* OR “long QT syndrome”  OR “sudden death*” OR “sudden cardiac death*” OR “ventricular tachycardia*” OR “ventricular fibrillation*” OR ECG OR electrocardiogra* OR “electrocardiogram pattern”)

#3 (“paralytic ileus” OR “intestinal obstruct*” OR “intestinal pseudo-obstruct” OR “burst abdom*” OR peritonitis OR sepsis OR agranulocytosis OR hypotens* OR “diabetic ketoacid*” OR DKA OR “neuroleptic malignant syndrome*” OR seizure* OR embol* OR thromb* OR “hepatic encephalopath*” OR “pericardial effusion*” OR obes* OR pancreatitis OR angina OR “myocardial infarc*” OR “acute coronary syndrome*” OR “coronary artery disease*” OR endocarditis OR pericarditis OR myocarditis OR cardiomyopath* OR “cardiac conduction system disease*” OR cardiomegal* OR cardiotox* OR “heart arrest*” OR “heart failure” OR “renal failure” OR “acute kidney injury” OR “renal insufficiency” OR “cardio-renal syndrome” OR rhabdomyolysis)

#1 AND #2 AND #3

All Searches were conducted on **13 October 2025**

| Database | Number of Articles |
| --- | --- |
| Medline Ovid | 228 |
| Embase | 2,802 |
| Psycinfo via EBSCOhost | 102 |
| Web of Science | 145 |
| Scopus | 4,882 |
| Total before Duplicates | 8159 |
| Total After Removing Duplicates on Endnote | 6,097 |
| Total Duplicates Removed | 2,062 |

### **Medline via Ovid using MeSH Terms**

Ovid MEDLINE(R) and Epub Ahead of Print, In-Process, In-Data-Review & Other Non-Indexed Citations, Daily and Versions <1946 to October 13, 2025>

1 Clozapine/ 9931

2 clozapine.mp. [mp=title, book title, abstract, original title, name of substance word, subject heading word, floating sub-heading word, keyword heading word, organism supplementary concept word, protocol supplementary concept word, rare disease supplementary concept word, unique identifier, synonyms, population supplementary concept word, anatomy supplementary concept word] 15792

3 1 or 2 15792

4 QT.mp. [mp=title, book title, abstract, original title, name of substance word, subject heading word, floating sub-heading word, keyword heading word, organism supplementary concept word, protocol supplementary concept word, rare disease supplementary concept word, unique identifier, synonyms, population supplementary concept word, anatomy supplementary concept word] 28208

5 QTc.mp. [mp=title, book title, abstract, original title, name of substance word, subject heading word, floating sub-heading word, keyword heading word, organism supplementary concept word, protocol supplementary concept word, rare disease supplementary concept word, unique identifier, synonyms, population supplementary concept word, anatomy supplementary concept word] 10822

6 exp Arrhythmias, Cardiac/ 251639

7 arrhythmia*.mp. [mp=title, book title, abstract, original title, name of substance word, subject heading word, floating sub-heading word, keyword heading word, organism supplementary concept word, protocol supplementary concept word, rare disease supplementary concept word, unique identifier, synonyms, population supplementary concept word, anatomy supplementary concept word] 171244

8 6 or 7 312688

9 Torsades de Pointes/ 2801

10 torsad*.mp. [mp=title, book title, abstract, original title, name of substance word, subject heading word, floating sub-heading word, keyword heading word, organism supplementary concept word, protocol supplementary concept word, rare disease supplementary concept word, unique identifier, synonyms, population supplementary concept word, anatomy supplementary concept word] 6014

11 9 or 10 6014

12 Long QT Syndrome/ 9349

13 "long QT syndrome*".mp. [mp=title, book title, abstract, original title, name of substance word, subject heading word, floating sub-heading word, keyword heading word, organism supplementary concept word, protocol supplementary concept word, rare disease supplementary concept word, unique identifier, synonyms, population supplementary concept word, anatomy supplementary concept word] 12027

14 12 or 13 12027

15 exp Death, Sudden/ 40033

16 "sudden death*".mp. [mp=title, book title, abstract, original title, name of substance word, subject heading word, floating sub-heading word, keyword heading word, organism supplementary concept word, protocol supplementary concept word, rare disease supplementary concept word, unique identifier, synonyms, population supplementary concept word, anatomy supplementary concept word] 27481

17 15 or 16 55679

18 exp Death, Sudden, Cardiac/ 19196

19 "sudden cardiac death*".mp. [mp=title, book title, abstract, original title, name of substance word, subject heading word, floating sub-heading word, keyword heading word, organism supplementary concept word, protocol supplementary concept word, rare disease supplementary concept word, unique identifier, synonyms, population supplementary concept word, anatomy supplementary concept word] 22758

20 18 or 19 32701

21 Tachycardia, Ventricular/ 17835

22 "ventricular tachycardia*".mp. [mp=title, book title, abstract, original title, name of substance word, subject heading word, floating sub-heading word, keyword heading word, organism supplementary concept word, protocol supplementary concept word, rare disease supplementary concept word, unique identifier, synonyms, population supplementary concept word, anatomy supplementary concept word] 30561

23 21 or 22 36587

24 Electrocardiography/ 207777

25 electrocardiogra*.mp. [mp=title, book title, abstract, original title, name of substance word, subject heading word, floating sub-heading word, keyword heading word, organism supplementary concept word, protocol supplementary concept word, rare disease supplementary concept word, unique identifier, synonyms, population supplementary concept word, anatomy supplementary concept word] 262997

26 24 or 25 262997

27 "electrocardiogram pattern".mp. [mp=title, book title, abstract, original title, name of substance word, subject heading word, floating sub-heading word, keyword heading word, organism supplementary concept word, protocol supplementary concept word, rare disease supplementary concept word, unique identifier, synonyms, population supplementary concept word, anatomy supplementary concept word] 186

28 4 or 5 or 8 or 11 or 14 or 17 or 20 or 23 or 26 or 27 529710

29 "paralytic ileus*".mp. [mp=title, book title, abstract, original title, name of substance word, subject heading word, floating sub-heading word, keyword heading word, organism supplementary concept word, protocol supplementary concept word, rare disease supplementary concept word, unique identifier, synonyms, population supplementary concept word, anatomy supplementary concept word] 1514

30 exp Intestinal Obstruction/ 52161

31 "intestinal obstruct*".mp. [mp=title, book title, abstract, original title, name of substance word, subject heading word, floating sub-heading word, keyword heading word, organism supplementary concept word, protocol supplementary concept word, rare disease supplementary concept word, unique identifier, synonyms, population supplementary concept word, anatomy supplementary concept word] 40574

32 30 or 31 59462

33 Intestinal Pseudo-Obstruction/ 2558

34 "intestinal pseudo-obstruct*".mp. [mp=title, book title, abstract, original title, name of substance word, subject heading word, floating sub-heading word, keyword heading word, organism supplementary concept word, protocol supplementary concept word, rare disease supplementary concept word, unique identifier, synonyms, population supplementary concept word, anatomy supplementary concept word] 3155

35 33 or 34 3155

36 "burst abdom*".mp. [mp=title, book title, abstract, original title, name of substance word, subject heading word, floating sub-heading word, keyword heading word, organism supplementary concept word, protocol supplementary concept word, rare disease supplementary concept word, unique identifier, synonyms, population supplementary concept word, anatomy supplementary concept word] 211

37 Peritonitis/ 26995

38 peritonitis.mp. [mp=title, book title, abstract, original title, name of substance word, subject heading word, floating sub-heading word, keyword heading word, organism supplementary concept word, protocol supplementary concept word, rare disease supplementary concept word, unique identifier, synonyms, population supplementary concept word, anatomy supplementary concept word] 46595

39 37 or 38 46595

40 exp Sepsis/ 153047

41 sepsis.mp. [mp=title, book title, abstract, original title, name of substance word, subject heading word, floating sub-heading word, keyword heading word, organism supplementary concept word, protocol supplementary concept word, rare disease supplementary concept word, unique identifier, synonyms, population supplementary concept word, anatomy supplementary concept word] 170154

42 40 or 41 234543

43 Agranulocytosis/ 7877

44 agranulocytosis.mp. [mp=title, book title, abstract, original title, name of substance word, subject heading word, floating sub-heading word, keyword heading word, organism supplementary concept word, protocol supplementary concept word, rare disease supplementary concept word, unique identifier, synonyms, population supplementary concept word, anatomy supplementary concept word] 9604

45 43 or 44 9604

46 exp Hypotension/ 31342

47 hypotens*.mp. [mp=title, book title, abstract, original title, name of substance word, subject heading word, floating sub-heading word, keyword heading word, organism supplementary concept word, protocol supplementary concept word, rare disease supplementary concept word, unique identifier, synonyms, population supplementary concept word, anatomy supplementary concept word] 97447

48 46 or 47 97802

49 Diabetic Ketoacidosis/ 8118

50 "diabetic ketoacid*".mp. [mp=title, book title, abstract, original title, name of substance word, subject heading word, floating sub-heading word, keyword heading word, organism supplementary concept word, protocol supplementary concept word, rare disease supplementary concept word, unique identifier, synonyms, population supplementary concept word, anatomy supplementary concept word] 12340

51 49 or 50 12340

52 DKA.mp. [mp=title, book title, abstract, original title, name of substance word, subject heading word, floating sub-heading word, keyword heading word, organism supplementary concept word, protocol supplementary concept word, rare disease supplementary concept word, unique identifier, synonyms, population supplementary concept word, anatomy supplementary concept word] 3984

53 Neuroleptic Malignant Syndrome/ 2205

54 "neuroleptic malignant syndrome*".mp. [mp=title, book title, abstract, original title, name of substance word, subject heading word, floating sub-heading word, keyword heading word, organism supplementary concept word, protocol supplementary concept word, rare disease supplementary concept word, unique identifier, synonyms, population supplementary concept word, anatomy supplementary concept word] 2997

55 53 or 54 2997

56 exp Seizures/ 78877

57 seizure*.mp. [mp=title, book title, abstract, original title, name of substance word, subject heading word, floating sub-heading word, keyword heading word, organism supplementary concept word, protocol supplementary concept word, rare disease supplementary concept word, unique identifier, synonyms, population supplementary concept word, anatomy supplementary concept word] 182286

58 56 or 57 186483

59 Embolism/ 14471

60 embol*.mp. [mp=title, book title, abstract, original title, name of substance word, subject heading word, floating sub-heading word, keyword heading word, organism supplementary concept word, protocol supplementary concept word, rare disease supplementary concept word, unique identifier, synonyms, population supplementary concept word, anatomy supplementary concept word] 215569

61 59 or 60 215569

62 Thrombosis/ 83074

63 thromb*.mp. [mp=title, book title, abstract, original title, name of substance word, subject heading word, floating sub-heading word, keyword heading word, organism supplementary concept word, protocol supplementary concept word, rare disease supplementary concept word, unique identifier, synonyms, population supplementary concept word, anatomy supplementary concept word] 630939

64 62 or 63 630939

65 Hepatic Encephalopathy/ 11727

66 "hepatic encephalopath*".mp. [mp=title, book title, abstract, original title, name of substance word, subject heading word, floating sub-heading word, keyword heading word, organism supplementary concept word, protocol supplementary concept word, rare disease supplementary concept word, unique identifier, synonyms, population supplementary concept word, anatomy supplementary concept word] 17052

67 65 or 66 17052

68 Pericardial Effusion/ 9679

69 "pericardial effusion*".mp. [mp=title, book title, abstract, original title, name of substance word, subject heading word, floating sub-heading word, keyword heading word, organism supplementary concept word, protocol supplementary concept word, rare disease supplementary concept word, unique identifier, synonyms, population supplementary concept word, anatomy supplementary concept word] 17740

70 68 or 69 17740

71 exp Obesity/ 287935

72 obes*.mp. [mp=title, book title, abstract, original title, name of substance word, subject heading word, floating sub-heading word, keyword heading word, organism supplementary concept word, protocol supplementary concept word, rare disease supplementary concept word, unique identifier, synonyms, population supplementary concept word, anatomy supplementary concept word] 504836

73 71 or 72 507183

74 exp Pancreatitis/ 59448

75 pancreatitis.mp. [mp=title, book title, abstract, original title, name of substance word, subject heading word, floating sub-heading word, keyword heading word, organism supplementary concept word, protocol supplementary concept word, rare disease supplementary concept word, unique identifier, synonyms, population supplementary concept word, anatomy supplementary concept word] 84234

76 74 or 75 84234

77 exp Angina, Stable/ or Angina Pectoris/ 35161

78 angina.mp. [mp=title, book title, abstract, original title, name of substance word, subject heading word, floating sub-heading word, keyword heading word, organism supplementary concept word, protocol supplementary concept word, rare disease supplementary concept word, unique identifier, synonyms, population supplementary concept word, anatomy supplementary concept word] 77848

79 77 or 78 77848

80 exp Myocardial Infarction/ 203236

81 "myocardial infarc*".mp. [mp=title, book title, abstract, original title, name of substance word, subject heading word, floating sub-heading word, keyword heading word, organism supplementary concept word, protocol supplementary concept word, rare disease supplementary concept word, unique identifier, synonyms, population supplementary concept word, anatomy supplementary concept word] 303244

82 80 or 81 309938

83 exp Acute Coronary Syndrome/ 22202

84 "acute coronary syndrome*".mp. [mp=title, book title, abstract, original title, name of substance word, subject heading word, floating sub-heading word, keyword heading word, organism supplementary concept word, protocol supplementary concept word, rare disease supplementary concept word, unique identifier, synonyms, population supplementary concept word, anatomy supplementary concept word] 49248

85 83 or 84 49248

86 exp Coronary Artery Disease/ 83376

87 "coronary artery disease*".mp. [mp=title, book title, abstract, original title, name of substance word, subject heading word, floating sub-heading word, keyword heading word, organism supplementary concept word, protocol supplementary concept word, rare disease supplementary concept word, unique identifier, synonyms, population supplementary concept word, anatomy supplementary concept word] 159540

88 86 or 87 159540

89 Endocarditis/ 12603

90 endocarditis.mp. [mp=title, book title, abstract, original title, name of substance word, subject heading word, floating sub-heading word, keyword heading word, organism supplementary concept word, protocol supplementary concept word, rare disease supplementary concept word, unique identifier, synonyms, population supplementary concept word, anatomy supplementary concept word] 49099

91 89 or 90 49099

92 Pericarditis/ 9558

93 pericarditis.mp. [mp=title, book title, abstract, original title, name of substance word, subject heading word, floating sub-heading word, keyword heading word, organism supplementary concept word, protocol supplementary concept word, rare disease supplementary concept word, unique identifier, synonyms, population supplementary concept word, anatomy supplementary concept word] 18769

94 92 or 93 18769

95 Myocarditis/ 18360

96 myocarditis.mp. [mp=title, book title, abstract, original title, name of substance word, subject heading word, floating sub-heading word, keyword heading word, organism supplementary concept word, protocol supplementary concept word, rare disease supplementary concept word, unique identifier, synonyms, population supplementary concept word, anatomy supplementary concept word] 29560

97 95 or 96 29560

98 exp Cardiomyopathies/ 120999

99 cardiomyopath*.mp. [mp=title, book title, abstract, original title, name of substance word, subject heading word, floating sub-heading word, keyword heading word, organism supplementary concept word, protocol supplementary concept word, rare disease supplementary concept word, unique identifier, synonyms, population supplementary concept word, anatomy supplementary concept word] 131103

100 98 or 99 167341

101 exp Cardiac Conduction System Disease/ 104168

102 "cardiac conduction system disease*".mp. [mp=title, book title, abstract, original title, name of substance word, subject heading word, floating sub-heading word, keyword heading word, organism supplementary concept word, protocol supplementary concept word, rare disease supplementary concept word, unique identifier, synonyms, population supplementary concept word, anatomy supplementary concept word] 2290

103 101 or 102 104207

104 Cardiomegaly/ 25605

105 cardiomegal*.mp. [mp=title, book title, abstract, original title, name of substance word, subject heading word, floating sub-heading word, keyword heading word, organism supplementary concept word, protocol supplementary concept word, rare disease supplementary concept word, unique identifier, synonyms, population supplementary concept word, anatomy supplementary concept word] 28886

106 104 or 105 28886

107 Cardiotoxicity/ 5971

108 cardiotox*.mp. [mp=title, book title, abstract, original title, name of substance word, subject heading word, floating sub-heading word, keyword heading word, organism supplementary concept word, protocol supplementary concept word, rare disease supplementary concept word, unique identifier, synonyms, population supplementary concept word, anatomy supplementary concept word] 24353

109 107 or 108 24353

110 exp Heart Arrest/ 60182

111 "heart arrest*".mp. [mp=title, book title, abstract, original title, name of substance word, subject heading word, floating sub-heading word, keyword heading word, organism supplementary concept word, protocol supplementary concept word, rare disease supplementary concept word, unique identifier, synonyms, population supplementary concept word, anatomy supplementary concept word] 42188

112 110 or 111 67966

113 exp Heart Failure/ 162038

114 "heart failure".mp. [mp=title, book title, abstract, original title, name of substance word, subject heading word, floating sub-heading word, keyword heading word, organism supplementary concept word, protocol supplementary concept word, rare disease supplementary concept word, unique identifier, synonyms, population supplementary concept word, anatomy supplementary concept word] 292546

115 113 or 114 293838

116 exp Renal Insufficiency/ 221286

117 ("renal failure" or "acute kidney injury" or "renal insufficiency" or "cardio-renal syndrome").mp. [mp=title, book title, abstract, original title, name of substance word, subject heading word, floating sub-heading word, keyword heading word, organism supplementary concept word, protocol supplementary concept word, rare disease supplementary concept word, unique identifier, synonyms, population supplementary concept word, anatomy supplementary concept word] 231867

118 116 or 117 304188

119 Rhabdomyolysis/ 6521

120 rhabdomyolysis.mp. [mp=title, book title, abstract, original title, name of substance word, subject heading word, floating sub-heading word, keyword heading word, organism supplementary concept word, protocol supplementary concept word, rare disease supplementary concept word, unique identifier, synonyms, population supplementary concept word, anatomy supplementary concept word] 11657

121 119 or 120 11657

122 29 or 32 or 35 or 36 or 39 or 42 or 45 or 48 or 51 or 52 or 55 or 58 or 61 or 64 or 67 or 70 or 73 or 76 or 79 or 82 or 85 or 88 or 91 or 94 or 97 or 100 or 103 or 106 or 109 or 112 or 115 or 118 or 121 3122927

123 3 and 28 and 122 228

### **EMBASE Search Strategy via Ovid using MeSH Terms**

Embase <1996 to 2025 Week 41>

1 Clozapine/ 35694

2 clozapine.mp. [mp=title, abstract, heading word, drug trade name, original title, device manufacturer, drug manufacturer, device trade name, keyword heading word, floating subheading word, candidate term word] 37994

3 1 or 2 37994

4 QT.mp. [mp=title, abstract, heading word, drug trade name, original title, device manufacturer, drug manufacturer, device trade name, keyword heading word, floating subheading word, candidate term word] 61769

5 QTc.mp. [mp=title, abstract, heading word, drug trade name, original title, device manufacturer, drug manufacturer, device trade name, keyword heading word, floating subheading word, candidate term word] 22529

6 exp Arrhythmias, Cardiac/ 679413

7 arrhythmia*.mp. [mp=title, abstract, heading word, drug trade name, original title, device manufacturer, drug manufacturer, device trade name, keyword heading word, floating subheading word, candidate term word] 229431

8 6 or 7 700380

9 Torsades de Pointes/ 9390

10 torsad*.mp. [mp=title, abstract, heading word, drug trade name, original title, device manufacturer, drug manufacturer, device trade name, keyword heading word, floating subheading word, candidate term word] 11214

11 9 or 10 11214

12 Long QT Syndrome/ 13530

13 "long QT syndrome*".mp. [mp=title, abstract, heading word, drug trade name, original title, device manufacturer, drug manufacturer, device trade name, keyword heading word, floating subheading word, candidate term word] 16229

14 12 or 13 16229

15 exp Death, Sudden/ 75510

16 "sudden death*".mp. [mp=title, abstract, heading word, drug trade name, original title, device manufacturer, drug manufacturer, device trade name, keyword heading word, floating subheading word, candidate term word] 51260

17 15 or 16 85337

18 exp Death, Sudden, Cardiac/ 30115

19 "sudden cardiac death*".mp. [mp=title, abstract, heading word, drug trade name, original title, device manufacturer, drug manufacturer, device trade name, keyword heading word, floating subheading word, candidate term word] 48095

20 18 or 19 48200

21 Tachycardia, Ventricular/ 51757

22 "ventricular tachycardia*".mp. [mp=title, abstract, heading word, drug trade name, original title, device manufacturer, drug manufacturer, device trade name, keyword heading word, floating subheading word, candidate term word] 41481

23 21 or 22 62563

24 Electrocardiography/ 138966

25 electrocardiogra*.mp. [mp=title, abstract, heading word, drug trade name, original title, device manufacturer, drug manufacturer, device trade name, keyword heading word, floating subheading word, candidate term word] 323754

26 24 or 25 323754

27 "electrocardiogram pattern".mp. [mp=title, abstract, heading word, drug trade name, original title, device manufacturer, drug manufacturer, device trade name, keyword heading word, floating subheading word, candidate term word] 222

28 4 or 5 or 8 or 11 or 14 or 17 or 20 or 23 or 26 or 27 930511

29 "paralytic ileus*".mp. [mp=title, abstract, heading word, drug trade name, original title, device manufacturer, drug manufacturer, device trade name, keyword heading word, floating subheading word, candidate term word] 4100

30 exp Intestinal Obstruction/ 102403

31 "intestinal obstruct*".mp. [mp=title, abstract, heading word, drug trade name, original title, device manufacturer, drug manufacturer, device trade name, keyword heading word, floating subheading word, candidate term word] 15153

32 30 or 31 105140

33 Intestinal Pseudo-Obstruction/ 2495

34 "intestinal pseudo-obstruct*".mp. [mp=title, abstract, heading word, drug trade name, original title, device manufacturer, drug manufacturer, device trade name, keyword heading word, floating subheading word, candidate term word] 1729

35 33 or 34 2845

36 "burst abdom*".mp. [mp=title, abstract, heading word, drug trade name, original title, device manufacturer, drug manufacturer, device trade name, keyword heading word, floating subheading word, candidate term word] 335

37 Peritonitis/ 36878

38 peritonitis.mp. [mp=title, abstract, heading word, drug trade name, original title, device manufacturer, drug manufacturer, device trade name, keyword heading word, floating subheading word, candidate term word] 64653

39 37 or 38 64653

40 exp Sepsis/ 350517

41 sepsis.mp. [mp=title, abstract, heading word, drug trade name, original title, device manufacturer, drug manufacturer, device trade name, keyword heading word, floating subheading word, candidate term word] 279501

42 40 or 41 388707

43 Agranulocytosis/ 8058

44 agranulocytosis.mp. [mp=title, abstract, heading word, drug trade name, original title, device manufacturer, drug manufacturer, device trade name, keyword heading word, floating subheading word, candidate term word] 8779

45 43 or 44 8779

46 exp Hypotension/ 176713

47 hypotens*.mp. [mp=title, abstract, heading word, drug trade name, original title, device manufacturer, drug manufacturer, device trade name, keyword heading word, floating subheading word, candidate term word] 198932

48 46 or 47 211994

49 Diabetic Ketoacidosis/ 19730

50 "diabetic ketoacid*".mp. [mp=title, abstract, heading word, drug trade name, original title, device manufacturer, drug manufacturer, device trade name, keyword heading word, floating subheading word, candidate term word] 21302

51 49 or 50 21302

52 DKA.mp. [mp=title, abstract, heading word, drug trade name, original title, device manufacturer, drug manufacturer, device trade name, keyword heading word, floating subheading word, candidate term word] 8741

53 Neuroleptic Malignant Syndrome/ 4391

54 "neuroleptic malignant syndrome*".mp. [mp=title, abstract, heading word, drug trade name, original title, device manufacturer, drug manufacturer, device trade name, keyword heading word, floating subheading word, candidate term word] 4615

55 53 or 54 4615

56 exp Seizures/ 226487

57 seizure*.mp. [mp=title, abstract, heading word, drug trade name, original title, device manufacturer, drug manufacturer, device trade name, keyword heading word, floating subheading word, candidate term word] 286065

58 56 or 57 290061

59 Embolism/ 23448

60 embol*.mp. [mp=title, abstract, heading word, drug trade name, original title, device manufacturer, drug manufacturer, device trade name, keyword heading word, floating subheading word, candidate term word] 335870

61 59 or 60 335870

62 Thrombosis/ 141774

63 thromb*.mp. [mp=title, abstract, heading word, drug trade name, original title, device manufacturer, drug manufacturer, device trade name, keyword heading word, floating subheading word, candidate term word] 1133658

64 62 or 63 1133658

65 Hepatic Encephalopathy/ 24678

66 "hepatic encephalopath*".mp. [mp=title, abstract, heading word, drug trade name, original title, device manufacturer, drug manufacturer, device trade name, keyword heading word, floating subheading word, candidate term word] 26851

67 65 or 66 26851

68 Pericardial Effusion/ 39518

69 "pericardial effusion*".mp. [mp=title, abstract, heading word, drug trade name, original title, device manufacturer, drug manufacturer, device trade name, keyword heading word, floating subheading word, candidate term word] 42577

70 68 or 69 42577

71 exp Obesity/ 736443

72 obes*.mp. [mp=title, abstract, heading word, drug trade name, original title, device manufacturer, drug manufacturer, device trade name, keyword heading word, floating subheading word, candidate term word] 809536

73 71 or 72 869305

74 exp Pancreatitis/ 112646

75 pancreatitis.mp. [mp=title, abstract, heading word, drug trade name, original title, device manufacturer, drug manufacturer, device trade name, keyword heading word, floating subheading word, candidate term word] 121971

76 74 or 75 121971

77 exp Angina, Stable/ or Angina Pectoris/ 64255

78 angina.mp. [mp=title, abstract, heading word, drug trade name, original title, device manufacturer, drug manufacturer, device trade name, keyword heading word, floating subheading word, candidate term word] 102877

79 77 or 78 102877

80 exp Myocardial Infarction/ 435981

81 "myocardial infarc*".mp. [mp=title, abstract, heading word, drug trade name, original title, device manufacturer, drug manufacturer, device trade name, keyword heading word, floating subheading word, candidate term word] 338229

82 80 or 81 476183

83 exp Acute Coronary Syndrome/ 87224

84 "acute coronary syndrome*".mp. [mp=title, abstract, heading word, drug trade name, original title, device manufacturer, drug manufacturer, device trade name, keyword heading word, floating subheading word, candidate term word] 104949

85 83 or 84 104949

86 exp Coronary Artery Disease/ 409066

87 "coronary artery disease*".mp. [mp=title, abstract, heading word, drug trade name, original title, device manufacturer, drug manufacturer, device trade name, keyword heading word, floating subheading word, candidate term word] 275719

88 86 or 87 436377

89 Endocarditis/ 22241

90 endocarditis.mp. [mp=title, abstract, heading word, drug trade name, original title, device manufacturer, drug manufacturer, device trade name, keyword heading word, floating subheading word, candidate term word] 59321

91 89 or 90 59321

92 Pericarditis/ 18108

93 pericarditis.mp. [mp=title, abstract, heading word, drug trade name, original title, device manufacturer, drug manufacturer, device trade name, keyword heading word, floating subheading word, candidate term word] 24169

94 92 or 93 24169

95 Myocarditis/ 37810

96 myocarditis.mp. [mp=title, abstract, heading word, drug trade name, original title, device manufacturer, drug manufacturer, device trade name, keyword heading word, floating subheading word, candidate term word] 46318

97 95 or 96 46318

98 exp Cardiomyopathies/ 245695

99 cardiomyopath*.mp. [mp=title, abstract, heading word, drug trade name, original title, device manufacturer, drug manufacturer, device trade name, keyword heading word, floating subheading word, candidate term word] 204222

100 98 or 99 269357

101 exp Cardiac Conduction System Disease/ 119906

102 "cardiac conduction system disease*".mp. [mp=title, abstract, heading word, drug trade name, original title, device manufacturer, drug manufacturer, device trade name, keyword heading word, floating subheading word, candidate term word] 83

103 101 or 102 119932

104 Cardiomegaly/ 16622

105 cardiomegal*.mp. [mp=title, abstract, heading word, drug trade name, original title, device manufacturer, drug manufacturer, device trade name, keyword heading word, floating subheading word, candidate term word] 17882

106 104 or 105 17882

107 Cardiotoxicity/ 48340

108 cardiotox*.mp. [mp=title, abstract, heading word, drug trade name, original title, device manufacturer, drug manufacturer, device trade name, keyword heading word, floating subheading word, candidate term word] 56505

109 107 or 108 56505

110 exp Heart Arrest/ 140605

111 "heart arrest*".mp. [mp=title, abstract, heading word, drug trade name, original title, device manufacturer, drug manufacturer, device trade name, keyword heading word, floating subheading word, candidate term word] 95718

112 110 or 111 140987

113 exp Heart Failure/ 704728

114 "heart failure".mp. [mp=title, abstract, heading word, drug trade name, original title, device manufacturer, drug manufacturer, device trade name, keyword heading word, floating subheading word, candidate term word] 549150

115 113 or 114 761243

116 exp Renal Insufficiency/ 556733

117 ("renal failure" or "acute kidney injury" or "renal insufficiency" or "cardio-renal syndrome").mp. [mp=title, abstract, heading word, drug trade name, original title, device manufacturer, drug manufacturer, device trade name, keyword heading word, floating subheading word, candidate term word] 224083

118 116 or 117 592735

119 Rhabdomyolysis/ 20457

120 rhabdomyolysis.mp. [mp=title, abstract, heading word, drug trade name, original title, device manufacturer, drug manufacturer, device trade name, keyword heading word, floating subheading word, candidate term word] 22297

121 119 or 120 22297

122 29 or 32 or 35 or 36 or 39 or 42 or 45 or 48 or 51 or 52 or 55 or 58 or 61 or 64 or 67 or 70 or 73 or 76 or 79 or 82 or 85 or 88 or 91 or 94 or 97 or 100 or 103 or 106 or 109 or 112 or 115 or 118 or 121 4788208

123 3 and 28 and 122 2802

124 Clozapine/ 35694

125 clozapine.mp. [mp=title, abstract, heading word, drug trade name, original title, device manufacturer, drug manufacturer, device trade name, keyword heading word, floating subheading word, candidate term word] 37994

126 124 or 125 37994

127 QT.mp. [mp=title, abstract, heading word, drug trade name, original title, device manufacturer, drug manufacturer, device trade name, keyword heading word, floating subheading word, candidate term word] 61769

128 QTc.mp. [mp=title, abstract, heading word, drug trade name, original title, device manufacturer, drug manufacturer, device trade name, keyword heading word, floating subheading word, candidate term word] 22529

129 exp Arrhythmias, Cardiac/ 679413

130 arrhythmia*.mp. [mp=title, abstract, heading word, drug trade name, original title, device manufacturer, drug manufacturer, device trade name, keyword heading word, floating subheading word, candidate term word] 229431

131 129 or 130 700380

132 Torsades de Pointes/ 9390

133 torsad*.mp. [mp=title, abstract, heading word, drug trade name, original title, device manufacturer, drug manufacturer, device trade name, keyword heading word, floating subheading word, candidate term word] 11214

134 132 or 133 11214

135 Long QT Syndrome/ 13530

136 "long QT syndrome*".mp. [mp=title, abstract, heading word, drug trade name, original title, device manufacturer, drug manufacturer, device trade name, keyword heading word, floating subheading word, candidate term word] 16229

137 135 or 136 16229

138 exp Death, Sudden/ 75510

139 "sudden death*".mp. [mp=title, abstract, heading word, drug trade name, original title, device manufacturer, drug manufacturer, device trade name, keyword heading word, floating subheading word, candidate term word] 51260

140 138 or 139 85337

141 exp Death, Sudden, Cardiac/ 30115

142 "sudden cardiac death*".mp. [mp=title, abstract, heading word, drug trade name, original title, device manufacturer, drug manufacturer, device trade name, keyword heading word, floating subheading word, candidate term word] 48095

143 141 or 142 48200

144 Tachycardia, Ventricular/ 51757

145 "ventricular tachycardia*".mp. [mp=title, abstract, heading word, drug trade name, original title, device manufacturer, drug manufacturer, device trade name, keyword heading word, floating subheading word, candidate term word] 41481

146 144 or 145 62563

147 Electrocardiography/ 138966

148 electrocardiogra*.mp. [mp=title, abstract, heading word, drug trade name, original title, device manufacturer, drug manufacturer, device trade name, keyword heading word, floating subheading word, candidate term word] 323754

149 147 or 148 323754

150 "electrocardiogram pattern".mp. [mp=title, abstract, heading word, drug trade name, original title, device manufacturer, drug manufacturer, device trade name, keyword heading word, floating subheading word, candidate term word] 222

151 127 or 128 or 131 or 134 or 137 or 140 or 143 or 146 or 149 or 150 930511

152 "paralytic ileus*".mp. [mp=title, abstract, heading word, drug trade name, original title, device manufacturer, drug manufacturer, device trade name, keyword heading word, floating subheading word, candidate term word] 4100

153 exp Intestinal Obstruction/ 102403

154 "intestinal obstruct*".mp. [mp=title, abstract, heading word, drug trade name, original title, device manufacturer, drug manufacturer, device trade name, keyword heading word, floating subheading word, candidate term word] 15153

155 153 or 154 105140

156 Intestinal Pseudo-Obstruction/ 2495

157 "intestinal pseudo-obstruct*".mp. [mp=title, abstract, heading word, drug trade name, original title, device manufacturer, drug manufacturer, device trade name, keyword heading word, floating subheading word, candidate term word] 1729

158 156 or 157 2845

159 "burst abdom*".mp. [mp=title, abstract, heading word, drug trade name, original title, device manufacturer, drug manufacturer, device trade name, keyword heading word, floating subheading word, candidate term word] 335

160 Peritonitis/ 36878

161 peritonitis.mp. [mp=title, abstract, heading word, drug trade name, original title, device manufacturer, drug manufacturer, device trade name, keyword heading word, floating subheading word, candidate term word] 64653

162 160 or 161 64653

163 exp Sepsis/ 350517

164 sepsis.mp. [mp=title, abstract, heading word, drug trade name, original title, device manufacturer, drug manufacturer, device trade name, keyword heading word, floating subheading word, candidate term word] 279501

165 163 or 164 388707

166 Agranulocytosis/ 8058

167 agranulocytosis.mp. [mp=title, abstract, heading word, drug trade name, original title, device manufacturer, drug manufacturer, device trade name, keyword heading word, floating subheading word, candidate term word] 8779

168 166 or 167 8779

169 exp Hypotension/ 176713

170 hypotens*.mp. [mp=title, abstract, heading word, drug trade name, original title, device manufacturer, drug manufacturer, device trade name, keyword heading word, floating subheading word, candidate term word] 198932

171 169 or 170 211994

172 Diabetic Ketoacidosis/ 19730

173 "diabetic ketoacid*".mp. [mp=title, abstract, heading word, drug trade name, original title, device manufacturer, drug manufacturer, device trade name, keyword heading word, floating subheading word, candidate term word] 21302

174 172 or 173 21302

175 DKA.mp. [mp=title, abstract, heading word, drug trade name, original title, device manufacturer, drug manufacturer, device trade name, keyword heading word, floating subheading word, candidate term word] 8741

176 Neuroleptic Malignant Syndrome/ 4391

177 "neuroleptic malignant syndrome*".mp. [mp=title, abstract, heading word, drug trade name, original title, device manufacturer, drug manufacturer, device trade name, keyword heading word, floating subheading word, candidate term word] 4615

178 176 or 177 4615

179 exp Seizures/ 226487

180 seizure*.mp. [mp=title, abstract, heading word, drug trade name, original title, device manufacturer, drug manufacturer, device trade name, keyword heading word, floating subheading word, candidate term word] 286065

181 179 or 180 290061

182 Embolism/ 23448

183 embol*.mp. [mp=title, abstract, heading word, drug trade name, original title, device manufacturer, drug manufacturer, device trade name, keyword heading word, floating subheading word, candidate term word] 335870

184 182 or 183 335870

185 Thrombosis/ 141774

186 thromb*.mp. [mp=title, abstract, heading word, drug trade name, original title, device manufacturer, drug manufacturer, device trade name, keyword heading word, floating subheading word, candidate term word] 1133658

187 185 or 186 1133658

188 Hepatic Encephalopathy/ 24678

189 "hepatic encephalopath*".mp. [mp=title, abstract, heading word, drug trade name, original title, device manufacturer, drug manufacturer, device trade name, keyword heading word, floating subheading word, candidate term word] 26851

190 188 or 189 26851

191 Pericardial Effusion/ 39518

192 "pericardial effusion*".mp. [mp=title, abstract, heading word, drug trade name, original title, device manufacturer, drug manufacturer, device trade name, keyword heading word, floating subheading word, candidate term word] 42577

193 191 or 192 42577

194 exp Obesity/ 736443

195 obes*.mp. [mp=title, abstract, heading word, drug trade name, original title, device manufacturer, drug manufacturer, device trade name, keyword heading word, floating subheading word, candidate term word] 809536

196 194 or 195 869305

197 exp Pancreatitis/ 112646

198 pancreatitis.mp. [mp=title, abstract, heading word, drug trade name, original title, device manufacturer, drug manufacturer, device trade name, keyword heading word, floating subheading word, candidate term word] 121971

199 197 or 198 121971

200 exp Angina, Stable/ or Angina Pectoris/ 64255

201 angina.mp. [mp=title, abstract, heading word, drug trade name, original title, device manufacturer, drug manufacturer, device trade name, keyword heading word, floating subheading word, candidate term word] 102877

202 200 or 201 102877

203 exp Myocardial Infarction/ 435981

204 "myocardial infarc*".mp. [mp=title, abstract, heading word, drug trade name, original title, device manufacturer, drug manufacturer, device trade name, keyword heading word, floating subheading word, candidate term word] 338229

205 203 or 204 476183

206 exp Acute Coronary Syndrome/ 87224

207 "acute coronary syndrome*".mp. [mp=title, abstract, heading word, drug trade name, original title, device manufacturer, drug manufacturer, device trade name, keyword heading word, floating subheading word, candidate term word] 104949

208 206 or 207 104949

209 exp Coronary Artery Disease/ 409066

210 "coronary artery disease*".mp. [mp=title, abstract, heading word, drug trade name, original title, device manufacturer, drug manufacturer, device trade name, keyword heading word, floating subheading word, candidate term word] 275719

211 209 or 210 436377

212 Endocarditis/ 22241

213 endocarditis.mp. [mp=title, abstract, heading word, drug trade name, original title, device manufacturer, drug manufacturer, device trade name, keyword heading word, floating subheading word, candidate term word] 59321

214 212 or 213 59321

215 Pericarditis/ 18108

216 pericarditis.mp. [mp=title, abstract, heading word, drug trade name, original title, device manufacturer, drug manufacturer, device trade name, keyword heading word, floating subheading word, candidate term word] 24169

217 215 or 216 24169

218 Myocarditis/ 37810

219 myocarditis.mp. [mp=title, abstract, heading word, drug trade name, original title, device manufacturer, drug manufacturer, device trade name, keyword heading word, floating subheading word, candidate term word] 46318

220 218 or 219 46318

221 exp Cardiomyopathies/ 245695

222 cardiomyopath*.mp. [mp=title, abstract, heading word, drug trade name, original title, device manufacturer, drug manufacturer, device trade name, keyword heading word, floating subheading word, candidate term word] 204222

223 221 or 222 269357

224 exp Cardiac Conduction System Disease/ 119906

225 "cardiac conduction system disease*".mp. [mp=title, abstract, heading word, drug trade name, original title, device manufacturer, drug manufacturer, device trade name, keyword heading word, floating subheading word, candidate term word] 83

226 224 or 225 119932

227 Cardiomegaly/ 16622

228 cardiomegal*.mp. [mp=title, abstract, heading word, drug trade name, original title, device manufacturer, drug manufacturer, device trade name, keyword heading word, floating subheading word, candidate term word] 17882

229 227 or 228 17882

230 Cardiotoxicity/ 48340

231 cardiotox*.mp. [mp=title, abstract, heading word, drug trade name, original title, device manufacturer, drug manufacturer, device trade name, keyword heading word, floating subheading word, candidate term word] 56505

232 230 or 231 56505

233 exp Heart Arrest/ 140605

234 "heart arrest*".mp. [mp=title, abstract, heading word, drug trade name, original title, device manufacturer, drug manufacturer, device trade name, keyword heading word, floating subheading word, candidate term word] 95718

235 233 or 234 140987

236 exp Heart Failure/ 704728

237 "heart failure".mp. [mp=title, abstract, heading word, drug trade name, original title, device manufacturer, drug manufacturer, device trade name, keyword heading word, floating subheading word, candidate term word] 549150

238 236 or 237 761243

239 exp Renal Insufficiency/ 556733

240 ("renal failure" or "acute kidney injury" or "renal insufficiency" or "cardio-renal syndrome").mp. [mp=title, abstract, heading word, drug trade name, original title, device manufacturer, drug manufacturer, device trade name, keyword heading word, floating subheading word, candidate term word] 224083

241 239 or 240 592735

242 Rhabdomyolysis/ 20457

243 rhabdomyolysis.mp. [mp=title, abstract, heading word, drug trade name, original title, device manufacturer, drug manufacturer, device trade name, keyword heading word, floating subheading word, candidate term word] 22297

244 242 or 243 22297

245 152 or 155 or 158 or 159 or 162 or 165 or 168 or 171 or 174 or 175 or 178 or 181 or 184 or 187 or 190 or 193 or 196 or 199 or 202 or 205 or 208 or 211 or 214 or 217 or 220 or 223 or 226 or 229 or 232 or 235 or 238 or 241 or 244 4788208

246 126 and 151 and 245 2802

### **Psycinfo via EBSCOhost**

102 Articles

| **Search #** | **Search Terms** | **Results** |
| --- | --- | --- |

| S1 | clozapine | 9,721 |
| --- | --- | --- |

| S2 | DE "Clozapine" | 6,880 |
| --- | --- | --- |

| S3 | (S1 OR S2) | 9,721 |
| --- | --- | --- |

| S4 | "QT" OR "QTc" | 1,543 |
| --- | --- | --- |

| S5 | DE "Heart Arrhythmias" | 1,330 |
| --- | --- | --- |

| S6 | arrhythmia* | 4,340 |
| --- | --- | --- |

| S7 | (S5 or S6) | 4,340 |
| --- | --- | --- |

| S8 | torsad* | 242 |
| --- | --- | --- |

| S9 | "long QT syndrome*" | 447 |
| --- | --- | --- |

| S10 | DE "Sudden Death" | 516 |
| --- | --- | --- |

| S11 | "sudden death*" | 1,473 |
| --- | --- | --- |

| S12 | (S10 or S11) | 1,473 |
| --- | --- | --- |

| S13 | "sudden cardiac death*" | 472 |
| --- | --- | --- |

| S14 | DE "Tachycardia" | 546 |
| --- | --- | --- |

| S15 | "ventricular tachycardia*" | 412 |
| --- | --- | --- |

| S16 | (S14 or S15) | 665 |
| --- | --- | --- |

| S17 | DE "Electrocardiography" | 4,245 |
| --- | --- | --- |

| S18 | electrocardiogra* | 6,381 |
| --- | --- | --- |

| S19 | (S17 or S18) | 6,381 |
| --- | --- | --- |

| S20 | "electrocardiogram pattern" | 3 |
| --- | --- | --- |

| S21 | (S4 OR S7 OR S8 OR S9 OR S12 OR S13 OR S16 OR S19 OR S20 OR S21) | 12,368 |
| --- | --- | --- |

| S22 | "paralytic ileus*" | 37 |
| --- | --- | --- |

| S23 | "intestinal obstruct*" | 95 |
| --- | --- | --- |

| S24 | "intestinal pseudo-obstruct*" | 33 |
| --- | --- | --- |

| S25 | "burst abdom*" | 0 |
| --- | --- | --- |

| S26 | peritonitis | 87 |
| --- | --- | --- |

| S27 | DE "Sepsis" | 576 |
| --- | --- | --- |

| S28 | sepsis | 1,411 |
| --- | --- | --- |

| S29 | S27 or S28 | 1,411 |
| --- | --- | --- |

| S30 | agranulocytosis | 693 |
| --- | --- | --- |

| S31 | DE "Hypotension" | 982 |
| --- | --- | --- |

| S32 | hypotens* | 2,973 |
| --- | --- | --- |

| S33 | (S31 or S32) | 2,973 |
| --- | --- | --- |

| S34 | "diabetic ketoacid*" or DKA | 222 |
| --- | --- | --- |

| S35 | DE "Neuroleptic Malignant Syndrome" | 1,097 |
| --- | --- | --- |

| S36 | "neuroleptic malignant syndrome*" | 1,348 |
| --- | --- | --- |

| S37 | (S35 or S36) | 1,348 |
| --- | --- | --- |

| S38 | DE "Seizures" | 17,494 |
| --- | --- | --- |

| S39 | seizure* | 40,135 |
| --- | --- | --- |

| S40 | (S38 or S39) | 40,135 |
| --- | --- | --- |

| S41 | DE "Embolisms" | 543 |
| --- | --- | --- |

| S42 | embol* | 2,272 |
| --- | --- | --- |

| S43 | (S41 or S42) | 2,272 |
| --- | --- | --- |

| S44 | DE "Thromboses" | 982 |
| --- | --- | --- |

| S45 | thromb* | 6,092 |
| --- | --- | --- |

| S46 | (S44 or S45) | 6,092 |
| --- | --- | --- |

| S47 | "hepatic encephalopath*" | 542 |
| --- | --- | --- |

| S48 | "pericardial effusion*" | 49 |
| --- | --- | --- |

| S49 | DE "Obesity" | 36,659 |
| --- | --- | --- |

| S50 | obes* | 58,215 |
| --- | --- | --- |

| S51 | (S49 or S50) | 58,215 |
| --- | --- | --- |

| S52 | pancreatitis | 442 |
| --- | --- | --- |

| S53 | DE "Angina Pectoris" | 543 |
| --- | --- | --- |

| S54 | angina | 1,458 |
| --- | --- | --- |

| S55 | S53 or S54 | 1,458 |
| --- | --- | --- |

| S56 | DE "Myocardial Infarctions" | 3,236 |
| --- | --- | --- |

| S57 | "myocardial infarc*" | 6,022 |
| --- | --- | --- |

| S58 | (S56 or S57) | 6,022 |
| --- | --- | --- |

| S59 | "acute coronary syndrome*" | 849 |
| --- | --- | --- |

| S60 | "coronary artery disease*" | 2,782 |
| --- | --- | --- |

| S61 | endocarditis | 235 |
| --- | --- | --- |

| S62 | pericarditis | 64 |
| --- | --- | --- |

| S63 | myocarditis | 305 |
| --- | --- | --- |

| S64 | (cardiomyopath*) OR (DE "Heart Disorders") | 12,585 |
| --- | --- | --- |

| S65 | "cardiac conduction system disease*" | 2 |
| --- | --- | --- |

| S66 | cardiomegal* | 74 |
| --- | --- | --- |

| S67 | cardiotox* | 308 |
| --- | --- | --- |

| S68 | "heart arrest*" | 455 |
| --- | --- | --- |

| S69 | "heart failure" | 5,489 |
| --- | --- | --- |

| S70 | ("renal failure" or "acute kidney injury" or "renal insufficiency" or "cardio-renal syndrome") | 1,976 |
| --- | --- | --- |

| S71 | rhabdomyolysis | 319 |
| --- | --- | --- |

| S72 | S22 OR S23 OR S24 OR S25 OR S26 OR S29 OR S30 OR S33 OR S34 OR S37 OR S40 OR S43 OR S46 OR S47 OR S48 OR S51 OR S52 OR S55 OR S58 OR S59 OR S60 OR S61 OR S62 OR S63 OR S64 OR S65 OR S66 OR S67 OR S68 OR S69 OR S70 OR S71 | 134,580 |
| --- | --- | --- |

| S73 | (S3 AND S21 AND S72) | 102 |
| --- | --- | --- |

### **Web of Science**

139 Articles

| **Search Number** | **Search Method** | **Number of Articles** |
| --- | --- | --- |
| 1 | **ALL=(clozapine)** | 21,298 |
| 2 | **ALL=((QT OR QTc OR arrhythmia* OR torsad* OR “long QT syndrome” OR “sudden death*” OR “sudden cardiac death*” OR “ventricular tachycardia*” OR “ventricular fibrillation*” OR ECG OR electrocardiogra* OR “electrocardiogram pattern”) )** | 402,815 |
| 3 | **ALL=((“paralytic ileus” OR “intestinal obstruct*" OR “intestinal pseudo-obstruct” OR “burst abdom*” OR peritonitis OR sepsis OR agranulocytosis OR hypotens* OR “diabetic ketoacid*” OR DKA OR “neuroleptic malignant syndrome*” OR seizure* OR embol* OR thromb* OR “hepatic encephalopath*” OR “pericardial effusion*” OR obes* OR pancreatitis OR angina OR “myocardial infarc*” OR “acute coronary syndrome*” OR “coronary artery disease*” OR endocarditis OR pericarditis OR myocarditis OR cardiomyopath* OR “cardiac conduction system disease*” OR cardiomegal* OR cardiotox* OR “heart arrest*” OR “heart failure” OR “renal failure” OR “acute kidney injury” OR “renal insufficiency” OR “cardio-renal syndrome” OR “rhabdomyolysis) )** | 3,152,298 |
| 4 | **#1 AND #2 AND #3** | 145 |

### **Scopus**

4,882 Articles

clozapine AND ( qt OR qtc OR arrhythmia* OR torsad* OR "long QT syndrome" OR "sudden death*" OR "sudden cardiac death*" OR "ventricular tachycardia*" OR "ventricular fibrillation*" OR ecg OR electrocardiogra* OR "electrocardiogram pattern" ) AND ( "paralytic ileus" OR "intestinal obstruct*" OR "intestinal pseudo-obstruct" OR "burst abdom*" OR peritonitis OR sepsis OR agranulocytosis OR hypotens* OR "diabetic ketoacid*" OR dka OR "neuroleptic malignant syndrome*" OR seizure* OR embol* OR thromb* OR "hepatic encephalopath*" OR "pericardial effusion*" OR obes* OR pancreatitis OR angina OR "myocardial infarc*" OR "acute coronary syndrome*" OR "coronary artery disease*" OR endocarditis OR pericarditis OR myocarditis OR cardiomyopath* OR "cardiac conduction system disease*" OR cardiomegal* OR cardiotox* OR "heart arrest*" OR "heart failure" OR "renal failure" OR "acute kidney injury" OR "renal insufficiency" OR "cardio-renal syndrome" OR rhabdomyolysis )

## **Search Criteria**

### Inclusion Criteria

- Focuses specifically on clozapine (other antipsychotics will not be included)
  - However, if it says antipsychotics more generally without excluding clozapine, then continue to full text
- Mention Sudden Cardiac Death or Sudden Death or may reference cardiac changes
  - Includes terms like sudden cardiac death, arrhythmias, QT prolongation, or other cardiac markers
- Can be any study type
- Can be any setting: hospital, community or geographic location
- Language: English
- Timeframe: does not matter

### Exclusion Criteria

- Unpublished studies
  - Cannot be a poster or conference abstract, must have a full text
- Non-human studies
- Sudden Death caused by Non-Cardiac Causes
- Overdose deaths
