# Supplementary material for: Risk factors for sudden cardiac death or sudden unexplained death in patients treated with clozapine: systematic review
Source: BJPsych Open. 2026 May 11;12(3):e130. doi: 10.1192/bjo.2026.11024 (PMC13169053; doi:10.1192/bjo.2026.11024)
Supplement: Easwar et al. supplementary material 2 — Easwar et al. supplementary material [file S2056472426110242sup002.pdf]

Supplementary Table 1 – Summary of ROBINS-E Assessments

Domain	Main Issues Identified Across Papers
1) Risk of Bias due to Confounding	• Incomplete or absent adjustment for key confounders, including illness severity, clozapine dose, and cardiometabolic risk factors.<br>• Some studies had no confounders included at all or relied on limited variables
2) Risk of Bias from Measurement of Exposure	• Exposure definition was generally clear, such as clozapine prescription or treatment status<br>• Time-varying exposures were not always measured, such as cumulative dose, plasma concentrations etc. This limited assessment of dose-response relationships.
3) Risk of Bias in Selection of Participants in the study	• Retrospective Study Designs often limited studies
4) Risk of Bias from Post-Exposure Interventions	• Differences in Clinical Monitoring between patients in some studies, such as increased surveillance in higher risk patients<br>• Changes of Clozapine Doses or discontinuations during follow-up were often not accounted for
5) Risk of Bias due to Missing Data	• Important confounders and exposure-related variables were often missing or unavailable, such as plasma clozapine levels, BMI, ECG findings, laboratory data.
6) Risk of Bias from Measurement of Outcomes	• Was generally rated well<br>• Outcome ascertainment was generally robust with many studies used death registries, medical records, and coroner reports with good information
7) Risk of Bias in Selection of the reported result	• Rated as low concern in all<br>• No evidence of multiple analyses or outcome-driven reporting
